# Room-temperature photosynthesis of propane from CO_2_ with Cu single atoms on vacancy-rich TiO_2_

**DOI:** 10.1038/s41467-023-36778-5

**Published:** 2023-02-27

**Authors:** Yan Shen, Chunjin Ren, Lirong Zheng, Xiaoyong Xu, Ran Long, Wenqing Zhang, Yong Yang, Yongcai Zhang, Yingfang Yao, Haoqiang Chi, Jinlan Wang, Qing Shen, Yujie Xiong, Zhigang Zou, Yong Zhou

**Affiliations:** 1 Key Laboratory of Modern Acoustics (MOE), Institute of Acoustics, School of Physics, Jiangsu Key Laboratory of Nanotechnology, Eco-materials and Renewable Energy Research Center (ERERC), National Laboratory of Solid State Microstructures, Collaborative Innovation Center of Advanced Microstructures, Nanjing University, Nanjing, China; 2 College of Engineering and Applied Sciences, Nanjing University, Nanjing, China; 3 School of Physics, Southeast University, Nanjing, China; 4 Institute of High Energy Physics, Chinese Academy of Sciences, Beijing, China; 5 Chemistry Interdisciplinary Research Center, School of Chemistry and Chemical Engineering, Yangzhou University, Yangzhou, China; 6 Hefei National Laboratory for Physical Sciences at the Microscale, Collaborative Innovation Center of Chemistry for Energy Materials (iChEM), School of Chemistry and Materials Science, University of Science and Technology of China, Hefei, China; 7 Key Laboratory of Soft Chemistry and Functional Materials (MOE), Nanjing University of Science and Technology, Nanjing, China; 8 School of Science and Engineering, the Chinese University of Hong Kong (Shenzhen), Shenzhen, China; 9 University of Electrocommunication, Graduate School of Informatics and Engineering, Chofu, Tokyo Japan; 10 School of Chemical and Environmental Engineering, Anhui Polytechnic University, Wuhu, China

**Keywords:** Renewable energy, Materials for energy and catalysis, Photocatalysis, Heterogeneous catalysis

## Abstract

Photochemical conversion of CO_2_ into high-value C_2+_ products is difficult to achieve due to the energetic and mechanistic challenges in forming multiple C-C bonds. Herein, an efficient photocatalyst for the conversion of CO_2_ into C_3_H_8_ is prepared by implanting Cu single atoms on Ti_0.91_O_2_ atomically-thin single layers. Cu single atoms promote the formation of neighbouring oxygen vacancies (V_O_s) in Ti_0.91_O_2_ matrix. These oxygen vacancies modulate the electronic coupling interaction between Cu atoms and adjacent Ti atoms to form a unique Cu-Ti-V_O_ unit in Ti_0.91_O_2_ matrix. A high electron-based selectivity of 64.8% for C_3_H_8_ (product-based selectivity of 32.4%), and 86.2% for total C_2+_ hydrocarbons (product-based selectivity of 50.2%) are achieved. Theoretical calculations suggest that Cu-Ti-V_O_ unit may stabilize the key *CHOCO and *CH_2_OCOCO intermediates and reduce their energy levels, tuning both C_1_-C_1_ and C_1_-C_2_ couplings into thermodynamically-favourable exothermal processes. Tandem catalysis mechanism and potential reaction pathway are tentatively proposed for C_3_H_8_ formation, involving an overall (**20**e^−^ – **20**H^+^) reduction and coupling of three CO_2_ molecules at room temperature.

## Abstract (web summary)

A photocatalyst for CO2 reduction to C3H8 is prepared by implanting Cu single atoms on vacancy rich TiO2 single layers. Key reaction intermediates, *CHOCO and *CH2OCOCO, are stabilized on the catalyst which promotes C-C bond formation.

## Introduction

Using sunlight to generate fuels from CO_2_ and water has the potential to reduce CO_2_ emissions and facilitate the large-scale storage of renewable energy^1–5^. Currently, light-driven reduction of CO_2_ is mainly limited to two-electron-reduced CO and further reduced C_1_ hydrocarbons such as methane (CH_4_)^6,7^ and C_2_ products such as ethene (C_2_H_4_)^8^ and ethane (C_2_H_6_)^9^ in few cases. However, the formation of C_3_ products by artificial photosynthesis is rare^10,11^ and generally relies on a higher-order reaction pathway that requires the sequential formation of multiple C-C bonds^12^, which involves the integration of two consecutive steps of CO_2_-to-CO and CO-to-C_2+_ at different catalytic centres^13–16^. These C-C couplings are usually challenging endothermic processes with huge uphill energy barriers owing to the high energy levels of the key *C_2_ and *C_3_ intermediates^17–20^. These energy barriers result from the lack of effective catalytic centres that can stabilize these multicarbon intermediates^21,22^.

Single-atom (SA) catalysts with maximum atom utilization efficiency and unique catalytic performance have emerged as an attractive frontier in heterogeneous catalysis^23,24^. Atomically thin two-dimensional (2D) materials are suitable platforms to anchor metal SAs^25,26^. These 2D single-layer (SL) materials can not only improve the activity of catalytic reactions^27^, but also provide ideal models to gain atomic-level insights into real active sites and reaction mechanisms of catalytic processes through experimental and theoretical techniques^25,28^.

Herein, we report that implanting Cu SAs in Ti_0.91_O_2_ atomic SLs allows the construction of a unique Cu-Ti-V_O_/Ti_0.91_O_2_-SL photocatalyst for highly efficient and selective conversion of CO_2_ into C_3_H_8_. The Cu SA promotes the neighboring Ti_0.91_O_2_ to generate oxygen vacancies (V_O_s) and form a Cu-Ti-V_O_ unit in the Ti_0.91_O_2_ matrix. The Cu-Ti-V_O_/Ti_0.91_O_2_-SL photocatalysis system efficiently converts CO_2_ into C_2+_ products at room temperature with high electron-based selectivity of 64.8% for C_3_H_8_ (product-based selectivity of 32.4%) and 86.2% for overall C_2+_ hydrocarbons (product-based selectivity of 50.2%). As suggested by theoretical calculations, the Cu-Ti-V_O_ units may stabilize the key *CHOCO and *CH_2_OCOCO intermediates and reduce their energy levels, tuning both C_1_-C_1_ and C_1_-C_2_ couplings into thermodynamically favorable exothermal processes. Based on the simulation results, tandem catalytic mechanism and reaction pathway for the production of C_2+_ hydrocarbons are proposed, involving an overall **20** e^—^**20** H^+^ reduction and two sequential C-C coupling processes of three CO_2_ molecules (**3** CO_2_ + **20** e^−^ + **20** H^+^→ C_3_H_8_ + **6** H_2_O). Our work provides an alternative paradigm for the photoconversion of CO_2_ into multicarbon solar fuels, and represents a progressive step toward imitating natural photosynthesis.

## Results

### Formation and characterization of Cu-Ti-V_O_ units

**Fig. 1:**
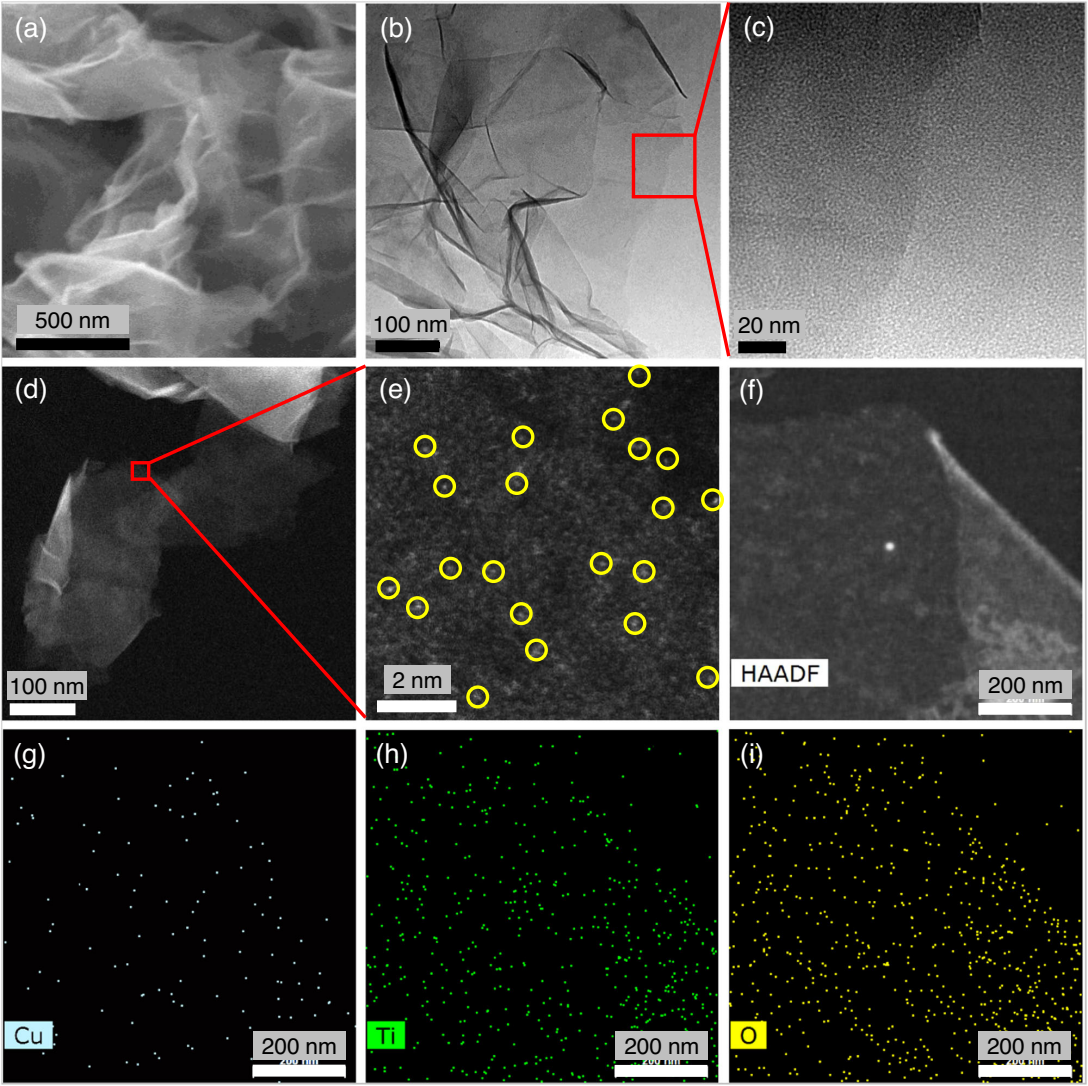
Morphological and structural characterization of Cu-Ti-V_O_/Ti_0.91_O_2_-SL. **a** FE-SEM and **b**, **c** TEM images, **d**, **e** AC HAADF-STEM images, and **f**–**i** EDS mapping.

Atomically thin layers of Ti_0.91_O_2_-SL were synthesized by exfoliating the layered protonic lepidocrocite-type titanate of H_0.7_Ti_1.825_O_4_^29,30^. The absence of peaks in the corresponding X-ray diffraction (XRD) pattern suggests the complete exfoliation into single layers^31^ (Supplementary Fig. 1). Cu SAs were then implanted in Ti_0.91_O_2_-SL to form Cu-Ti-V_O_/Ti_0.91_O_2_-SL through a modified wet-chemical route, followed by a rapid thermal treatment (RTT) in an Ar atmosphere^32^ (see Methods for details). The amount of Cu loading was detected to be 0.29 wt% by inductively coupled plasma–optical emission spectrometry (ICP-OES). The XRD pattern of Cu-Ti-V_O_/Ti_0.91_O_2_-SL was similar to that of pristine Ti_0.91_O_2_-SL (Supplementary Fig. 1). The thickness of Cu-Ti-V_O_/Ti_0.91_O_2_-SL was found to be 0.85 nm using atomic force microscopy (AFM) (Supplementary Fig. 2), which corresponded closely to the theoretical monolayer thickness (0.75 nm)^30,33,34^. Field emission-scanning electron microscopy (FE-SEM) and transmission electron microscopy (TEM) images show the ultrathin sheet-like morphology of Cu-Ti-V_O_/Ti_0.91_O_2_-SL without discernible nanoparticles (NPs) (Fig. 1a–c), potentially implying the atomic-scale size of the Cu embedded in the Ti_0.91_O_2_ matrix. The isolated bright dots in atomic resolution aberration-corrected high angle annular dark-field-scanning transmission electron microscopy (AC HADDF-STEM) images (Fig. 1e) directly confirm the atomic dispersion of the Cu in the matrix. Energy dispersive X-ray spectroscopy (EDS) mapping (Fig. 1f–i) indicates that Cu is evenly distributed throughout the atomically thin Ti_0.91_O_2_ matrix.

**Fig. 2:**
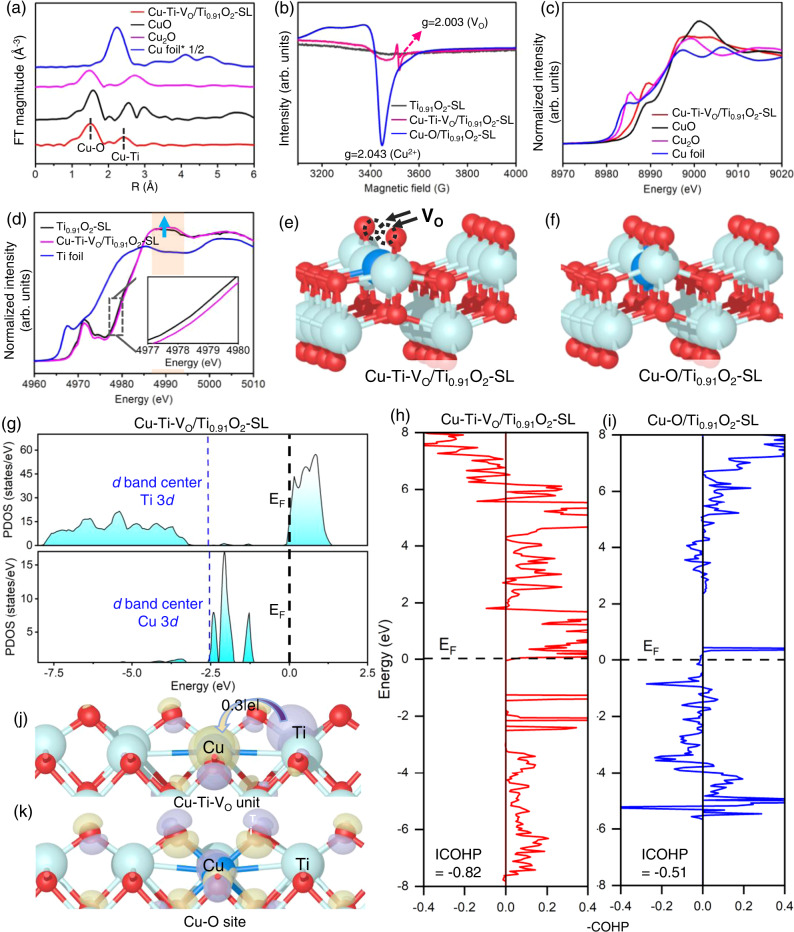
Electronic structure of Cu-Ti-V_O_/Ti_0.91_O_2_-SL. **a** Fourier transforms of EXAFS spectra at the Cu K-edge. **b** EPR spectra. Normalized XANES spectra at the **c** Cu K-edge and **d** Ti K-edge. **e**, **f** The atomic structure configuration (colour codes: light blue (Ti), blue (Cu), and red (O)). **g** PDOS and d-band centres of Cu 3*d* and Ti 3*d* orbitals. **h**, **i** COHP between Cu and adjacent Ti. **j**, **k** Charge density differences (yellow represents electron accumulation, and purple denotes electron depletion).

Fourier transforms of the Cu K-edge extended X-ray absorption fine structure (EXAFS) spectra of Cu-Ti-V_O_/Ti_0.91_O_2_-SL (Fig. 2a) contain a prominent peak at ~1.56 Å, assigned to Cu-O coordination in the first shell with a coordination number of 4 according to the EXAFS fitting analysis (Supplementary Fig. 3 and Supplementary Table 1). The characteristic metallic Cu-Cu bonding at ~2.24 Å is not observed, further validating the single-atom distribution of Cu. A minor scattering peak at ~2.42 Å is attributed to Cu-Ti coordination in the second shell based on the EXAFS fitting results^34,35^. The presence of Cu-Ti coordination, originating from the strong electronic interaction between Cu and adjacent Ti atoms, suggests that Cu is present in the atomically dispersed Cu-Ti dual-metal coordination form^36^.

The normalized Cu K-edge X-ray absorption near-edge structure (XANES) spectra show that the near-edge absorption energy of Cu-Ti-V_O_/Ti_0.91_O_2_-SL is higher than that of Cu foil and lies between the energies of Cu_2_O and CuO (Fig. 2c), indicating that the average oxidation state of Cu is between +1 and +2, which is verified by Cu LMM Auger electron spectra (AES) and Cu 2*p* X-ray photoelectron spectra (XPS) as well as the theoretical results (Supplementary Fig. 4a, c). The near-edge absorption energy and white-line intensity of Ti K-edge XANES for Cu-Ti-V_O_/Ti_0.91_O_2_-SL are higher than those for Ti_0.91_O_2_-SL (Fig. 2d), suggesting the presence of Ti species with lower electronic density, which is confirmed by the Ti^δ+^ (δ > 4) species in Ti 2*p* XPS spectra^37,38^ (Supplementary Fig. 4b). The formation of these electron-poor Ti centres and the partially oxidized Cu centres proves the electron donation to Cu SAs from the coordinated Ti atoms as a result of the strong electronic interaction in the Cu-Ti dual-metal coordination.

The O 1 *s* XPS spectrum of Cu-Ti-V_O_/Ti_0.91_O_2_-SL displays a peak attributed to V_O_ at 531.6 eV^39,40^ (Supplementary Fig. 5). The electron paramagnetic resonance (EPR) spectra also show a V_O_ signal at a g value of 2.003^40^ (Fig. 2b), suggesting the formation of V_O_s in Cu-Ti-V_O_/Ti_0.91_O_2_-SL. In contrast, no V_O_ signal is observed for Ti_0.91_O_2_-SL (Fig. 2b and Supplementary Fig. 5). Density functional theory (DFT) computations reveal that the formation energy of V_O_ is 5.66 eV in Ti_0.91_O_2_-SL, while the value sharply decreases to 2.39 eV with Cu anchoring, implying that the introduction of Cu SAs can facilitate the formation of neighboring V_O_s in the Ti_0.92_O_2_ matrix.

The strong coordination interaction of the anchored Cu SA with neighboring Ti atoms originates from the presence of V_O_s. A control sample without V_O_s (denoted as Cu-O/Ti_0.91_O_2_-SL) was synthesized through a similar RTT in an air atmosphere (see Methods, Fig. 2b, f and Supplementary Fig. 6 for details). The projected density of states (PDOS) of Cu-Ti-V_O_/Ti_0.91_O_2_-SL (Fig. 2g) demonstrates that the *d*-band centre (ɛ_d_) of Cu (−2.52 eV) is in good agreement with adjacent Ti (−2.58 eV), inducing a strong electronic coupling effect in the dual-metal sites^36^. The crystal orbital Hamilton population (COHP) between Cu SAs and the closest Ti atoms was then calculated to quantitatively study the intensity of Cu-Ti interactions with and without V_O_s. The less antibonding orbital populations and a more negative value of integrated-crystal orbital Hamilton population (ICOHP) for Cu-Ti-V_O_/Ti_0.91_O_2_-SL than Cu-O/Ti_0.91_O_2_-SL prove that the Cu-Ti electronic interaction is much stronger when V_O_s are present (Fig. 2h, i). Charge density differences and Bader charge analysis (Fig. 2j) suggest that 0.3 e^−^ directly transfers to Cu SAs from the neighboring Ti atoms in Cu-Ti-V_O_/Ti_0.91_O_2_-SL, which causes notable electron accumulation at Cu sites and substantial electron depletion at Ti sites, revealing the asymmetric electron distribution at Cu-Ti coordination. In comparison, without V_O_s, Cu-O/Ti_0.91_O_2_-SL displays no discernible electron perturbation at Ti sites, and electrons are localized within Cu-O coordination (Fig. 2k), demonstrating that Cu SAs share negligible interactions with Ti atoms and are in a relatively isolated single-metal form, confirmed by XPS and EPR (Fig. 2b and Supplementary Fig. 6). Therefore, both experimental and theoretical analyses indicate that Cu-Ti-V_O_ units are formed in the Ti_0.91_O_2_ matrix for Cu-Ti-V_O_/Ti_0.91_O_2_-SL. In contrast, isolated Cu-O sites are formed in the Ti_0.91_O_2_ matrix without V_O_s for Cu-O/Ti_0.91_O_2_-SL.

### Photocatalytic CO_2_ reduction performance

**Fig. 3:**
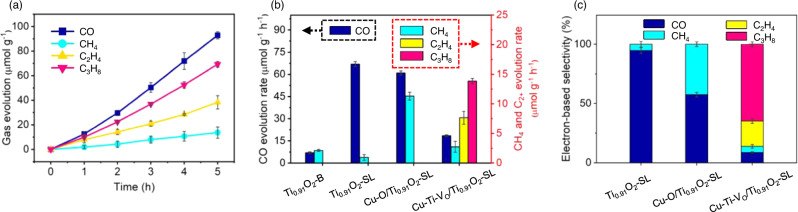
Photocatalytic CO_2_ reduction performance in acetonitrile aqueous solution. **a** Photocatalytic product evolution as a function of light irradiation times on Cu-Ti-V_O_/Ti_0.91_O_2_-SL. **b** Product formation rates and **c** electron-based selectivity on Ti_0.91_O_2_-SL, Cu-O/Ti_0.91_O_2_-SL, and Cu-Ti-V_O_/Ti_0.91_O_2_-SL. (Error bars indicate standard deviations.).

All of the photocatalytic CO_2_ reduction metrics reported in this study were measured in CO_2_-saturated acetonitrile aqueous solution (acetonitrile: water = 5:1 by volume) unless otherwise specified, and the testing details are described in the Methods section (Supplementary Fig. 7). Unexfoliated layered titanate (denoted as Ti_0.91_O_2_-B) mainly produces CO, with a formation rate of 7.0 μmol g^−1^ h^−1^ (Fig. 3b). Ti_0.91_O_2_-SL displays an enhanced CO production rate of 67.0 μmol g^−1^ h^−1^, indicating that the atomically thin 2D geometry is favorable for the improvement of CO_2_ activity through the potential exposure of many rich active sites and a shortened charge-transfer distance from the interior to the surface^26,41^. Both CO and CH_4_ were detected on Cu-O/Ti_0.91_O_2_-SL with yields of 61.0 and 11.3 μmol g^−1^ h^−1^, respectively. Cu-Ti-V_O_/Ti_0.91_O_2_-SL exhibit significant yields of C_2_H_4_ (7.6 μmol g^−1^h^−1^) and C_3_H_8_ (13.8 μmol g^−1^h^−1^), together with a small amount of CH_4_ and trace C_2_H_6_/C_3_H_6_, in addition to CO (18.6 μmolg^−1^h^−1^) (Fig. 3a, b), showing a strong capability of C-C coupling. No H_2_ production was detected (Supplementary Fig. 8), and O_2_ was generated roughly stoichiometrically (Supplementary Fig. 9). The quantum efficiency (QE) was obtained 0.48%, 0.15%, and 0.06% at the wavelength of 385, 415, and 520 nm, respectively. A high electron-based selectivity of C_3_H_8_ of 64.8% was achieved (32.4% of the product-based selectivity), and that of total C_2+_ products was as high as 86.2% (50.2% of the product-based selectivity) (Fig. 3c and Table 1). The photocatalytic performance of Cu-Ti-V_O_/Ti_0.91_O_2_-SL was also tested in pure water. The production rates of all carbon-based products decreased in pure water as compared with those (Supplementary Fig. 10). Moreover, the overall selectivity of C_2+_ products becomes lower in pure water, and the selectivity of C_3_H_8_ is less than C_2_H_4_ (Supplementary Fig. 11). H_2_ is detected with a formation rate of ~2.2 μmol g^−1^ h^−1^ in pure water. The improved activity and selectivity are most likely due to the high CO_2_ solubility in acetonitrile, which largely increases the local accessibility of CO_2_, thus enhancing the contact between the catalyst and CO_2_ molecules to facilitate C-C coupling. To the best of our knowledge, the efficiency and selectivity of C_3_H_8_ production in our work outperform most of the reported photocatalysts in acetonitrile medium, and also rank among the top of the reported C_2+_ production photocatalysts in non-acetonitrile medium (Supplementary Table 3).

**Table 1:** Selectivity of different products on Ti_0.91_O_2_-SL, Cu-O/Ti_0.91_O_2_-SL, and Cu-Ti-V_O_/Ti_0.91_O_2_-SL.^a^

Catalyst	Electron-based selectivity (%)				Product-based selectivity (%)			
	CO	CH_4_	C_2_H_4_	C_3_H_8_	CO	CH_4_	C_2_H_4_	C_3_H_8_
Ti_0.91_O_2_-SL	94.7	5.3	Not detectable		98.6	1.4	Not detectable	
Cu-O/Ti_0.91_O_2_-SL	57.4	42.6	Not detectable		84.4	15.6	Not detectable	
Cu-Ti-V_O_/Ti_0.91_O_2_-SL	8.7	5.1	21.4	64.8	43.4	6.3	17.8	32.4

^a^ The details regarding the calculation of electron- and product-based selectivity of CO, CH_4_, C_2_H_4_ and C_3_H_8_ are presented in the Methods section.

The substantially suppressed CO production and increased C_2+_ production on Cu-Ti-V_O_/Ti_0.91_O_2_-SL, compared with pristine Ti_0.91_O_2_-SL, imply that the formation of C_2+_ products is potentially derived from the coupling depletion of the *CO intermediate. CO was used to substitute CO_2_ as the starting reactant on Cu-Ti-V_O_/Ti_0.91_O_2_-SL, and a considerable amount of C_3_H_8_ and C_2_H_4_ was indeed detected (Supplementary Fig. 12), further confirming *CO as an important intermediate for the present C_2+_ products. The distinctive CO_2_ photoreduction activity and high selectivity of C_3_H_8_ and total C_2+_ products are still well maintained after three-cycle tests of 15 h in total (Supplementary Figs. 13 and 14), and the post-reaction characterizations of Cu-Ti-V_O_/Ti_0.91_O_2_-SL display no obvious structural and morphological changes (Supplementary Figs. 15–17), demonstrating the excellent stability of the catalyst. Notably, with the decrease in the Cu loading amount, the C_2+_ yield declines as the lower Cu loading reasonably reduces the number of Cu-Ti-V_O_ units, restraining C-C coupling (Supplementary Figs. 18 and 19). Moreover, higher Cu loading results in Cu aggregation into nanoclusters (NCs) and nanoparticles (NPs), subsequently lowering the selectivity of C_2+_ products, which is probably due to the weaker coupling interaction between Cu and the Ti_0.91_O_2_ matrix as the metal particle size increases^42,43^ (Supplementary Fig. 18). A series of control experiments were performed in the absence of illumination, the catalyst or CO_2_, and no detectable CO or other hydrocarbon products were detected. The ^13^CO_2_ isotope labeling experiment and the time profile of relative abundance of ^13^C labeled products confirm that the carbon source for CO and other hydrocarbon products originates from the input CO_2_ gas^44^ (Supplementary Figs. 20 and 21, and Supplementary Table 4).

The light utilization and the charge carrier dynamics of the as-prepared catalysts were studied. The absorption edge of single-layer Ti_0.91_O_2_-SL displays a blue shift compared with layered Ti_0.91_O_2_-B due to the quantum confinement effect of monolayer structure, while the light absorption is enhanced after implantation of Cu single atoms (Supplementary Fig. 22). Photoelectrochemical (PEC) measurements confirm the enhanced charge separation and migration efficiency of Cu-Ti-V_O_/Ti_0.91_O_2_-SL, ascribed to the 2D atomically-thin structure, which effectively shortens the charge transfer distance from body to surface and lowers charge recombination possibility (Supplementary Fig. 23). The fast charge carrier dynamics of Cu-Ti-V_O_/Ti_0.91_O_2_-SL is kinetically favorable for the multi-electron reactions of generating C_2+_ products. Moreover, CO_2_ adsorption isotherms reveal that Cu-Ti-V_O_/Ti_0.91_O_2_-SL possesses the highest CO_2_ uptake capacity, which is a priority for CO_2_ activation and reduction (Supplementary Fig. 24).

### Theoretical calculations for CO_2_ photoreduction mechanism

**Fig. 4:**
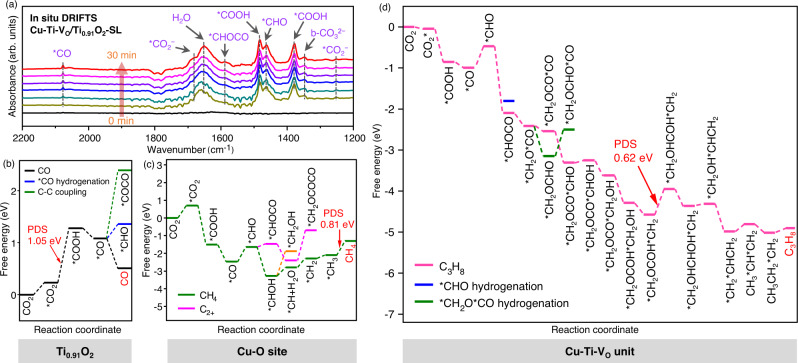
Mechanism studies of CO_2_ reduction on Cu-Ti-V_O_/Ti_0.91_O_2_-SL. **a** In situ DRIFTS spectra of the photocatalytic CO_2_ reduction on Cu-Ti-V_O_/Ti_0.91_O_2_-SL. Gibbs free energy diagrams of CO_2_ reduction on **b** Ti_0.91_O_2_ matrix, **c** Cu-O site, and **d** Cu-Ti-V_O_ unit.

The substantial suppression of CO production and promotion of C_2+_ production by Cu-Ti-V_O_/Ti_0.91_O_2_-SL, in sharp contrast to pristine Ti_0.91_O_2_-SL, has demonstrated the key role of Cu-Ti-V_O_ units in coupling *CO intermediates into C_2+_ products. In fact, our in situ diffuse reflectance Fourier transform infrared spectroscopy (DRIFTS) characterization using ^12^CO_2_ and ^13^CO_2_ has detected the formation of the key *COOH, *CO, *CHO, and *CHOCO intermediates on Cu-Ti-V_O_/Ti_0.91_O_2_-SL (Fig. 4a, Supplementary Figs. 25 and 26, and Supplementary Note 1), indicating the coupling of *CO into *CHOCO intermediates. However, it is not feasible to experimentally establish a direct spatial correlation between the active sites and the evolution of reaction intermediates to allow for deeper understanding on CO_2_ photoreduction mechanism on atomic level. For this reason, density functional theory (DFT) calculations with a computational hydrogen electrode (CHE) model^45,46^ was employed in an attempt to describe one potential mechanism for the reaction of CO_2_-to-C_2+_ products on Cu-Ti-V_O_/Ti_0.91_O_2_-SL.

In the calculations, Cu-Ti-V_O_/Ti_0.91_O_2_-SL are featured with two types of catalytic centres for CO_2_ reduction: the Cu atom-free Ti_0.91_O_2_ matrix domain and the Cu-Ti-V_O_ unit domain. On Ti_0.91_O_2_ domain, CO_2_ is reduced to *CO through *COOH intermediate^47^ (Fig. 4b and Supplementary Fig. 27). DFT results suggest the easy desorption of *CO on Ti_0.91_O_2_ matrix rather than further hydrogenation or C-C coupling (Supplementary Note 2), consistent with the experimental observation of the dominant CO product on pristine Ti_0.91_O_2_-SL. On Cu-Ti-V_O_ unit domain, the absorbed CO_2_ is firstly converted to *CHO through *COOH and *CO intermediates^48^ (Supplementary Fig. 27). Then, the *CHO at Cu-Ti-V_O_ unit may couple with the CO diffusing from neighboring Ti_0.91_O_2_ domain to generate *CHOCO^49–51^ with free energy change of −1.63 eV (see Supplementary Note 3 for more details about potential pathways). The following C_1_-C_2_ coupling (*CH_2_OCO + *CO → *CH_2_OCOCO) is also calculated to be a thermodynamically-favorable exergonic reaction (−0.13 eV) (Supplementary Note 3). As a contrast, it is worth noting that on the Cu-O site without Vos, C-C coupling processes are found to be challenging owing to the large uphill energy changes, while the hydrogenation of *CO into CH_4_ is more preferred (Fig. 4c)^52,53^. Meanwhile, for Cu-Ti-V_O_ unit, some of the *C_2_ species will continue to hydrogenate through a series of proton-electron steps to form C_2_H_4_. The free energy change of the potential determining step (PDS) is calculated as 0.62 eV for the C_3_H_8_ formation pathway (Fig. 4d) and 0.90 eV for the C_2_H_4_ formation pathway (Supplementary Fig. 28), suggesting the easier formation of C_3_H_8_ than C_2_H_4_. This is in accordance with our photocatalytic experimental observation of the higher yield of C_3_H_8_ than C_2_H_4_ on Cu-Ti-V_O_/Ti_0.91_O_2_-SL. The overall reaction pathway for the reduction of CO_2_ to C_3_H_8_ and C_2_H_4_ over Cu-Ti-V_O_/Ti_0.91_O_2_-SL is tentatively described in Supplementary Fig. 29. In summary, the above results suggest a tandem catalysis mechanism^13–16,54–56^, where the Cu-free Ti_0.91_O_2_ matrix may be preferential to participate in the reduction of CO_2_ to CO, and Cu-Ti-V_O_ unit is more beneficial to the exergonic C-C coupling to C_2+_ products (Supplementary Fig. 30).

The above result suggests favorable exergonic reactions for both C_1_-C_1_ and C_1_-C_2_ couplings on Cu-Ti-V_O_ unit, which is in sharp contrast with Cu-O sites and other previously-reported catalysts with challenging endergonic C-C couplings^17–20^. Such downhill energy changes of C-C coupling on Cu-Ti-V_O_ unit are probably owing to the low energy levels of the *CHOCO and *CH_2_OCOCO intermediates (Supplementary Fig. 31). A stable multiple-bonding configuration containing one Cu-C bond and two Ti-O bonds is built for the adsorption of *CHOCO on Cu-Ti-V_O_ unit (Supplementary Fig. 32). Moreover, a five-membered ring is formed between *CH_2_OCOCO and Cu-Ti-V_O_ unit, largely alleviating the electron accumulation and relaxing the intermolecular and intramolecular electrostatic repulsion (Supplementary Fig. 33). The electronic and geometric effect of Cu-Ti-V_O_ unit may jointly stabilize these key *C_2+_ intermediates and lower their adsorption energy levels to promote C-C couplings.

## Discussion

A unique Cu-Ti-V_O_ unit in atomically thin Ti_0.91_O_2_ monolayer nanosheets was developed for highly efficient and selective photoconversion of CO_2_ to C_3_H_8_. Theoretical calculations suggested that the Cu-Ti-V_O_ unit, as a favorable reaction centre for both C_1_-C_1_ and C_1_-C_2_ couplings, can effectively facilitate the multistep photocatalytic reduction of CO_2_ by reducing the energy levels of the key *CHOCO and *CH_2_OCOCO intermediates via electronic and geometric effects. A tandem mechanism and possible reaction pathway are proposed for the conversion of CO_2_ to C_3_H_8_. The results of our study may open an alternative avenue for designing and synthesizing tandem photocatalysts with dual-metal active sites and coordination vacancies that modulate the behavior of reaction intermediates for the production of multicarbon fuels driven by light.

## Methods

### Synthesis of catalysts

Cu-Ti-V_O_/Ti_0.91_O_2_-SL: Single-layer Ti_0.91_O_2_ nanosheets were synthesized according to previous literature^29^, and the specific procedure is presented below. The parent Cs compound, Cs_0.7_Ti_1.825_O_4,_ was obtained by repeating twice the heat treatment (800 °C, 20 h) for the mixture of 10 mmol Cs_2_CO_3_ and 53 mmol anatase TiO_2_. The interlayer Cs ions were extracted by stirring 5 g of the as-prepared Cs_0.7_Ti_1.825_O_4_ in 500 mL of 1 M HCl solution for 24 h. After four cycles of acid exchange, the solid was washed with DI water to remove excess acid and then dried in a freeze dryer. Layered protonic lepidocrocite-type titanate of H_0.7_Ti_1.825_O_4_ was obtained.

0.4 g of the as-prepared layered H_0.7_Ti_1.825_O_4_ was shaken with 100 mL of 0.08 M tetrabutylammonium (TBA) hydroxide aqueous solution for a week to produce stable colloidal suspensions of atomically thin Ti_0.91_O_2_ single layers. The colloidal suspensions were then dried in a freeze dryer.

The Cu-en precursor was prepared by mixing 30 mL of 0.025 g/L CuCl_2_‧2H_2_O aqueous solution with 360 μL of ethanediamine (en) at room temperature. Then, this Cu-en precursor was added to 7 g of the as-prepared colloidal suspensions of single-layer Ti_0.91_O_2_. After stirring for 5 h, the solid was filtrated, washed with deionized water, and dried in a freeze dryer to obtain a single-layer Cu-en/Ti_0.91_O_2_ sample. Then, the Cu-Ti-V_O_/Ti_0.91_O_2_-SL sample was prepared by rapid thermal treatment (RTT) of single-layer Cu-en/Ti_0.91_O_2_ in an Ar atmosphere at 500 °C for 1 min^32^. Briefly, the powders were put into a quartz tube, which was then inserted into a tube furnace preheated to 500 °C. Under Ar flow, the powders were kept at that temperature for 1 min, and then the quartz tube was quickly removed and rapidly cooled to room temperature.

Cu-O/Ti_0.91_O_2_-SL: The sample was prepared by the same method as Cu-Ti-V_O_/Ti_0.91_O_2_-SL, except that the RTT process was conducted in an air atmosphere.

Cu-Ti-V_O_/Ti_0.91_O_2_-SL (lower): The sample was prepared by the same method as Cu-Ti-V_O_/Ti_0.91_O_2_-SL, except that an aqueous solution of 0.0125 g/L CuCl_2_‧2H_2_O was used.

Cu NC/Ti_0.91_O_2_-SL: The sample was prepared by the same method as Cu-Ti-V_O_/Ti_0.91_O_2_-SL, except that an aqueous solution of 0.1 g/L CuCl_2_‧2H_2_O was used.

Cu NP/Ti_0.91_O_2_-SL: The sample was prepared by the same method as Cu-Ti-V_O_/Ti_0.91_O_2_-SL, except that an aqueous solution of 0.2 g/L CuCl_2_‧2H_2_O aqueous was used, and RTT was maintained at 10 min.

Ti_0.91_O_2_-B: The sample was prepared by treating layered protonic lepidocrocite-type titanate of H_0.7_Ti_1.825_O_4_ with the same RTT process as used for Cu-Ti-V_O_/Ti_0.91_O_2_-SL.

### Characterization

XRD data were measured on an X-ray diffractometer (Rigaku Ultima III, Japan) by Cu-Kα radiation (λ = 0.154178 nm) at 40 kV and 40 mA with a scan rate of 5° min^−1^. AFM analysis was performed on an MFP3D microscope (Asylum Research, MFP-3D-SA, USA) with a cantilever operating in tapping mode. The morphology was characterized by FE-SEM (FEI NOVA NANOSEM 230). TEM images were taken on an FEI Tecnai F20 TEM apparatus. Atomic-resolution STEM-HAADF images were obtained on a double spherical aberration-corrected STEM/TEM FEI Titan3 Cubed 60–300, and the samples were suspended on micron-scale carbon grids of Mo mesh. The Cu concentration was determined by ICP–OES with an Avio200 instrument. The XAFS spectra (Cu K-edge and Ti K-edge) were collected at the 1W1B station at the Beijing Synchrotron Radiation Facility (BSRF). The storage rings at BSRF were operated at 2.5 GeV with an average current of 250 mA. Using a Si (111) double-crystal monochromator, the data collection was conducted in transmission/fluorescence mode using an ionization chamber. All spectra were collected under ambient conditions. The EXAFS data were processed according to standard procedures using the ATHENA module implemented in the IFEFFIT software package. The k^3^-weighted EXAFS spectra were obtained by subtracting the postedge background from the overall absorption and then normalizing with respect to the edge jump step. Subsequently, k^3^-weighted χ(k) data of the Cu K-edge underwent Fourier transform to real (R) space using Hanning windows (dk = 1.0 Å^−1^) to separate the EXAFS contributions from different coordination shells. To obtain the quantitative structural parameters around the central atoms, least squares curve parameter fitting was performed using the ARTEMIS module of IFEFFIT software packages. The chemical states of the samples were detected by XPS and AES, which were equipped with an ultrahigh vacuum Thermo Fisher Scientific electron spectrometer by using Al Kα radiation (1486.6 eV) as the X-ray source, and the binding energies were calibrated according to the C 1 s peak of adventitious carbon at 284.6 eV. The in situ DRIFTS spectra were obtained on a Bruker IFS 66 v FT spectrometer with Harrick diffuse reflectance with ZnSe and quartz windows at BL01B in NSRL, Hefei.

### Measurements of photocatalytic CO_2_ reduction

Photocatalytic CO_2_ reduction experiments were performed in a Pyrex reaction vessel with a top irradiation window. Typically, 10 mg of photocatalyst powder was suspended in a 15 mL CO_2_-saturated solution containing 12.5 mL acetonitrile and 2.5 mL H_2_O in a 166 mL quartz reaction cell. Before illumination, the system was filled with CO_2_ (purity >99.999%) to 1 atm. The reactor was then irradiated by a 300 W Xe lamp (PerfectLight, PLS-SXE300). During irradiation, 0.1 mL of gas was collected from the reaction headspace every hour, and the gaseous products were analysed by using gas chromatography (GC-2014C, Shimadzu Corp., Japan). The isotope experiment was conducted using ^13^CO_2_ as feedstock, and the products were analysed using gas chromatography–mass spectrometry (7890 A and 5975 C, Agilent). The quantum efficiency was evaluated using a LED lamp (PerfectLight) with the wavelength of 385,415, or 520 nm as the light source.

The electron-based selectivity of C_3_H_8_ was calculated using (1):

1 $${{{{{{\rm{Sel}}}}}}}_{{{{{{\rm{electron}}}}}}}\left({{{{{{\rm{C}}}}}}}_{3}{{{{{{\rm{H}}}}}}}_{8}\right)=\left(\frac{n\,({{{{{{\rm{C}}}}}}}_{3}{{{{{{\rm{H}}}}}}}_{8})\times 20}{n({{{{{\rm{CO}}}}}})\times 2+n({{{{{\rm{C}}}}}}{{{{{{\rm{H}}}}}}}_{4})\times 8+n({{{{{{\rm{C}}}}}}}_{2}{{{{{{\rm{H}}}}}}}_{4})\times 12+n({{{{{{\rm{C}}}}}}}_{3}{{{{{{\rm{H}}}}}}}_{8})\times 20}\right)\times 100\%$$

The product-based selectivity of C_3_H_8_ was calculated using (2):

2 $${{{{{{\rm{Sel}}}}}}}_{{{{{{\rm{product}}}}}}}\left({{{{{{\rm{C}}}}}}}_{3}{{{{{{\rm{H}}}}}}}_{8}\right)=\left(\frac{n\left({{{{{{\rm{C}}}}}}}_{3}{{{{{{\rm{H}}}}}}}_{8}\right)}{n\left({{{{{\rm{CO}}}}}}\right)+n\left({{{{{\rm{C}}}}}}{{{{{{\rm{H}}}}}}}_{4}\right)+n\left({{{{{{\rm{C}}}}}}}_{2}{{{{{{\rm{H}}}}}}}_{4}\right)+n\left({{{{{{\rm{C}}}}}}}_{3}{{{{{{\rm{H}}}}}}}_{8}\right)}\right)\times 100\%$$

The electron-based selectivity of C_2+_ products was calculated using (3):

3 $${{{{{{\rm{Sel}}}}}}}_{{{{{{\rm{electron}}}}}}}\left({{{{{{\rm{C}}}}}}}_{2+}\right)=\left(\frac{n\left({{{{{{\rm{C}}}}}}}_{2}{{{{{{\rm{H}}}}}}}_{4}\right)\times 12+n\left({{{{{{\rm{C}}}}}}}_{3}{{{{{{\rm{H}}}}}}}_{8}\right)\times 20}{n\left({{{{{\rm{CO}}}}}}\right)\times 2+n\left({{{{{\rm{C}}}}}}{{{{{{\rm{H}}}}}}}_{4}\right)\times 8+n\left({{{{{{\rm{C}}}}}}}_{2}{{{{{{\rm{H}}}}}}}_{4}\right)\times 12+n\left({{{{{{\rm{C}}}}}}}_{3}{{{{{{\rm{H}}}}}}}_{8}\right)\times 20}\right)\times 100\%$$

The product-based selectivity of C_2+_ products was calculated using (4):

4 $${{{{{{\rm{Sel}}}}}}}_{{{{{{\rm{product}}}}}}}\left({{{{{{\rm{C}}}}}}}_{2+}\right)=\left(\frac{n({{{{{{\rm{C}}}}}}}_{2}{{{{{{\rm{H}}}}}}}_{4})+n({{{{{{\rm{C}}}}}}}_{3}{{{{{{\rm{H}}}}}}}_{8})}{n({{{{{\rm{CO}}}}}})+n({{{{{\rm{C}}}}}}{{{{{{\rm{H}}}}}}}_{4})+n({{{{{{\rm{C}}}}}}}_{2}{{{{{{\rm{H}}}}}}}_{4})+n({{{{{{\rm{C}}}}}}}_{3}{{{{{{\rm{H}}}}}}}_{8})}\right)\times 100\%$$

where *n* is the formation rate.

The quantum efficiency was calculated using (5):

5 $${{{{{\rm{QE}}}}}}=\frac{N\left({{{{{\rm{CO}}}}}}\right)\times 2+N\left({{{{{\rm{C}}}}}}{{{{{{\rm{H}}}}}}}_{4}\right)\times 8+N\left({{{{{{\rm{C}}}}}}}_{2}{{{{{{\rm{H}}}}}}}_{4}\right)\times 12+N\left({{{{{{\rm{C}}}}}}}_{3}{{{{{{\rm{H}}}}}}}_{8}\right)\times 20}{N\left({{{{{\rm{photon}}}}}}\right)}\times 100\%$$

where *N* is the number of evolved gas molecules or incident photons.

### Computational details

All calculations were performed based on density functional theory (DFT) through the Vienna ab initio Simulation Package (VASP)^57^. Projector-augmented-wave (PAW) pseudopotentials^58^ were used to treat the core electrons, while interactions between electrons were described by the Perdew-Burke-Ernzerhof (PBE)^59^ exchange-correlation functional of the generalized gradient approximation (GGA). For all the calculations, the vacuum space in the z-direction was 20 Å to avoid potential interactions between periodic surfaces. The DFT-D3 method of Grimme^60^ was applied to describe the van der Waals dispersion forces. DFT + *U* approach^61,62^ with *U* = 3.5 eV was considered to evaluate the influence of strongly correlated d electrons on the calculated free energies. According to our previous report^63^, the results of calculated free energies obtained from DFT + *U* and DFT are consistent. Therefore, regular DFT was employed in this work. The Monkhorst-Pack k-point grid with 3 × 3 × 1 mesh was applied until the maximal forces on each ion were smaller than 0.02 eV/Å. The convergence criterion of the energy was set to 10^−4^ eV, and a cut-off energy of 450 eV was used for the plane wave expansion. The Gibbs free energy change (Δ*G*) was defined as follows^45,64^:

6 $$\Delta G=\Delta E+\Delta {E}_{{{{{{\rm{ZPE}}}}}}}-T\Delta S$$

where Δ*E* is the energy difference between the reactants and product obtained through DFT calculations. Δ*E*_ZPE_ and Δ*S* are the changes in the zero-point energies (ZPE) and entropy^65^. *T* represents the temperature and was set as 298.15.

### Statistics & reproducibility

No statistical method was used to predetermine the sample size. No data were excluded from the analyses. The experiments were not randomized.

## Supplementary information

Supplementary Information

## Data Availability

The data that support the findings of this study are available within the paper and its supplementary information files or are available from the corresponding authors upon reasonable request. Source data are provided with this paper.

## Source data

Source data

## Code availability

All related codes regarding DFT simulations in this study are provided in the Source Data file.
