# Supplementary material for: Room-temperature photosynthesis of propane from CO2 with Cu single atoms on vacancy-rich TiO2
Source: Nat Commun. 2023 Feb 27;14:1117. doi: 10.1038/s41467-023-36778-5 (PMC9970977; doi:10.1038/s41467-023-36778-5)
Supplement: Supplementary file 1 — Supplementary Information [file 41467_2023_36778_MOESM1_ESM.pdf]

Supplementary Information

Room-Temperature Photosynthesis of Propane from CO_2 with Cu Single Atoms on Vacancy-rich TiO_2

Author list: Yan Shen[1,2,#], Chunjin Ren[3,#], Lirong Zheng[4], Xiaoyong Xu[5], Ran Long[6], Wenqing Zhang[6], Yong Yang[7], Yongcai Zhang[5], Yingfang Yao[1,2,8], Haoqiang Chi[1], Jinlan Wang[3]*, Qing Shen[9], Yujie Xiong[6]*, Zhigang Zou[1,2,8] and Yong Zhou[1,8,10]*

Affiliations:

[1]Key Laboratory of Modern Acoustics (MOE), Institute of Acoustics, School of Physics, Jiangsu Key Laboratory of Nanotechnology, Eco-materials and Renewable Energy Research Center (ERERC), National Laboratory of Solid State Microstructures, Collaborative Innovation Center of Advanced Microstructures, Nanjing University, Nanjing, China.

[2]College of Engineering and Applied Sciences, Nanjing University, Nanjing, China.

[3]School of Physics, Southeast University, Nanjing, China.

[4]Institute of High Energy Physics, Chinese Academy of Sciences, Beijing, China.

[5]Chemistry Interdisciplinary Research Center, School of Chemistry and Chemical Engineering, Yangzhou University, Yangzhou, China.

[6]Hefei National Laboratory for Physical Sciences at the Microscale, Collaborative Innovation Center of Chemistry for Energy Materials (iChEM), School of Chemistry and Materials Science, University of Science and Technology of China, Hefei, China.

[7]Key Laboratory of Soft Chemistry and Functional Materials (MOE), Nanjing University of Science and Technology, Nanjing, China.

[8]School of Science and Engineering, the Chinese University of Hong Kong (Shenzhen), Shenzhen, China.

[9]University of Electrocommunication, Graduate School of Informatics and Engineering, Chofu, Tokyo, Japan.

[10]School of Chemical and Environmental Engineering, Anhui Polytechnic University, Wuhu, China.

[#]These authors contributed to this work equally: Yan Shen and Chunjin Ren.

*Correspondence to: zhouyong1999@nju.edu.cn (Y. Z.); jlwang@seu.edu.cn (J. W.); yjxiong@ustc.edu.cn (Y. X.)

This Supplementary PDF file includes:

Supplementary Figures 1 to 33

Supplementary Tables 1 to 4

Supplementary Notes 1 to 4

Supplementary References 1 to 60

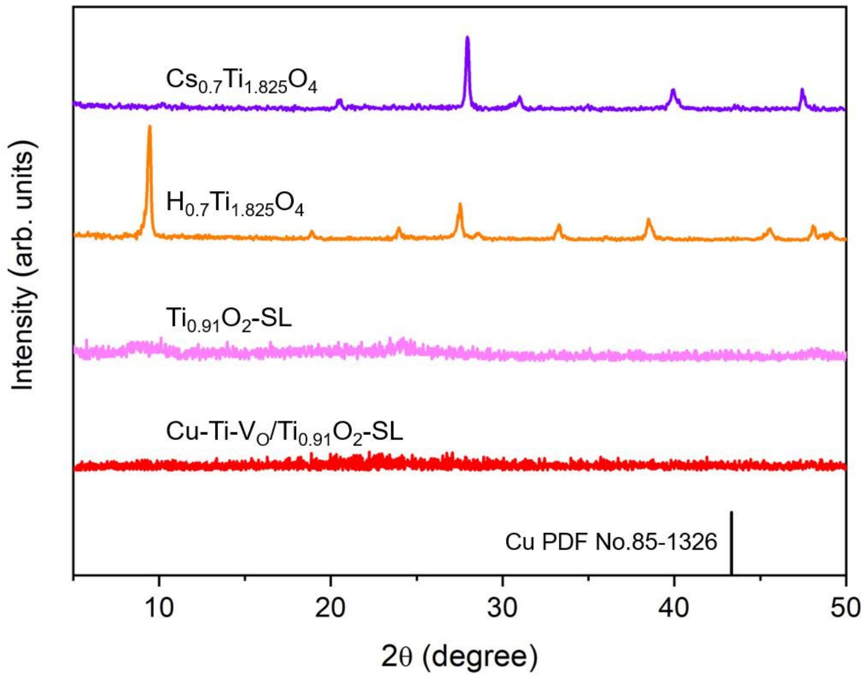

Supplementary Figure 1. XRD patterns for $Cs_{0.7}Ti_{1.825}O_4$, $H_{0.7}Ti_{1.825}O_4$, $Ti_{0.91}O_2$-SL, and Cu-Ti-V_O/$Ti_{0.91}O_2$-SL.

No distinct Bragg reflection is observed in the XRD patterns for exfoliated $Ti_{0.91}O_2$-SL, implying the disappearance of the periodic layered structure, and the complete exfoliation into single layers[1]. The XRD pattern for Cu-Ti-V_O/$Ti_{0.91}O_2$-SL is similar to $Ti_{0.91}O_2$-SL, indicating no restacking during Cu implanting process.

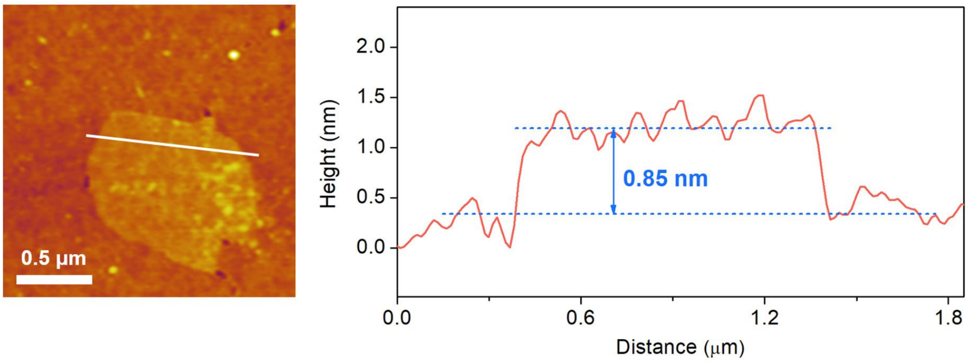

Supplementary Figure 2. AFM image of Cu-Ti-V_O/$Ti_{0.91}O_2$-SL and the corresponding high profile.

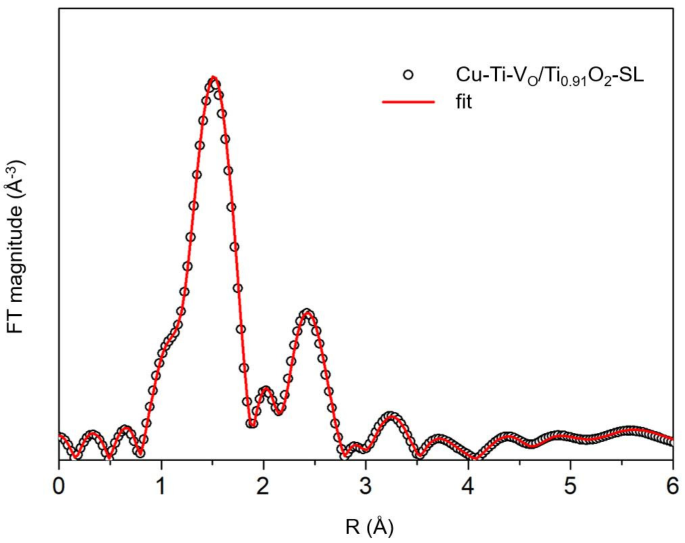

Supplementary Figure 3. EXAFS data and the fit for Cu-Ti-V_O/$Ti_{0.91}O_2$-SL in R-space. The gray dots are data and the red line is the fit.

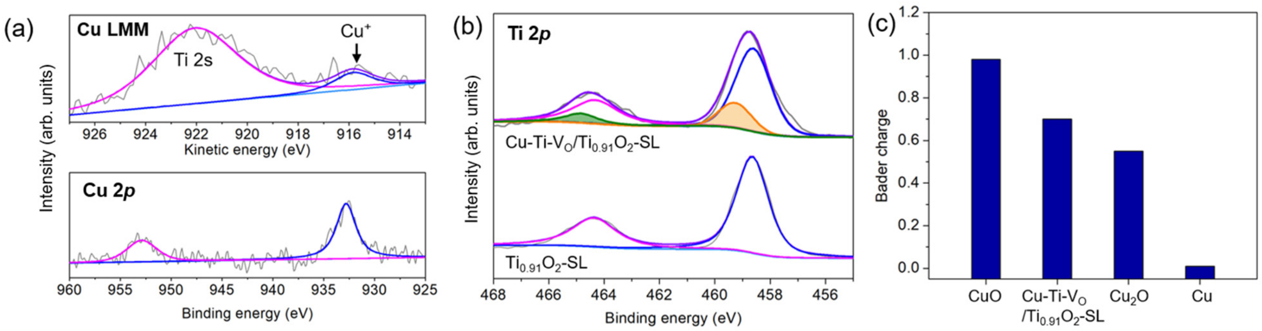

Supplementary Figure 4. (a) Cu LMM AES and Cu 2*p* XPS spectra of Cu-Ti-V_O/$Ti_{0.91}O_2$-SL. (b) Ti 2*p* of Cu-Ti-V_O/$Ti_{0.91}O_2$-SL and $Ti_{0.91}O_2$-SL with RTT process. (c) Bader charge analysis of Cu atoms in Cu-Ti-V_O/$Ti_{0.91}O_2$-SL and the references.

According to AES of Cu LMM for Cu-Ti-V_O/$Ti_{0.91}O_2$-SL, only peak for Cu^+ with the kinetic energy of 915.8 eV is detected, while the peak for Cu^0 at 918.3 eV is absent[2,3]. The Cu 2$p_{3/2}$ main peak locates at the binding energy of 932.8 eV, between those of Cu^+ (932.0 eV) and Cu^{2+} (934.0 eV), and the characteristic satellite peak of Cu^{2+} is absent in Cu 2*p* XPS spectrum[4,5]. These indicate that the Cu in Cu-Ti-V_O/$Ti_{0.91}O_2$-SL is in the oxidation state between +1 and +2. In addition, the theoretical results demonstrated that the Bader charge of Cu in Cu-Ti-V_O/$Ti_{0.91}O_2$-SL is 0.7e, which is higher than Cu_2O (0.55e), and less than CuO (0.98e), demonstrating the valance of Cu was between +1 and +2 in Cu-Ti-V_O/$Ti_{0.91}O_2$-SL, quite compatible with XPS and XANES analysis.

The 2$p_{3/2}$ and 2$p_{1/2}$ peaks in Ti 2*p* spectra of Cu-Ti-V_O/$Ti_{0.91}O_2$-SL are broadened and exhibit positive shift compared to the pristine $Ti_{0.91}O_2$-SL, and they can be fitted by introducing two new peaks at higher binding energy, which are ascribed to $Ti^{\delta+}$ ($\delta > 4$) species with less electron density[6], in line with the XANES results.

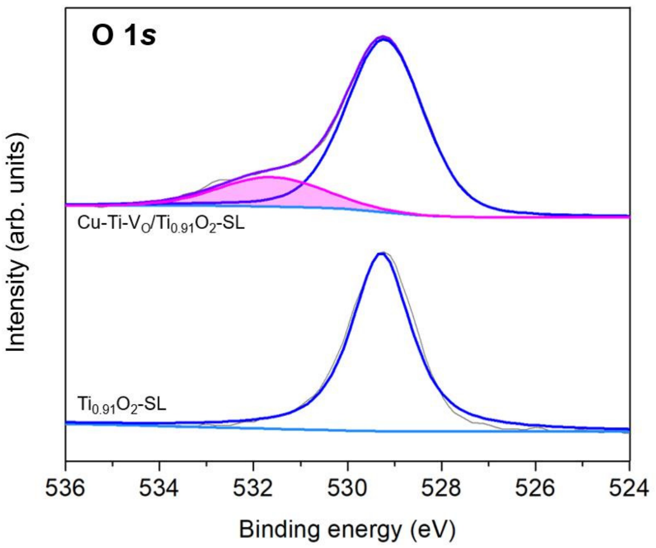

Supplementary Figure 5. O 1*s* XPS spectra of Cu-Ti-V_O/$Ti_{0.91}O_2$-SL and $Ti_{0.91}O_2$-SL with RTT process.

Two peaks are observed in the O 1*s* XPS spectrum of Cu-Ti-V_O/$Ti_{0.91}O_2$-SL: the peak at 529.3 eV is deemed as the lattice oxygen, and the other one located at 531.6 eV is attributed to V_O[7,8], suggesting the presence of V_O in Cu-Ti-V_O/$Ti_{0.91}O_2$-SL. Yet, only one peak assigned to the lattice oxygen is detected in $Ti_{0.91}O_2$-SL, demonstrating V_O is absent in $Ti_{0.91}O_2$-SL.

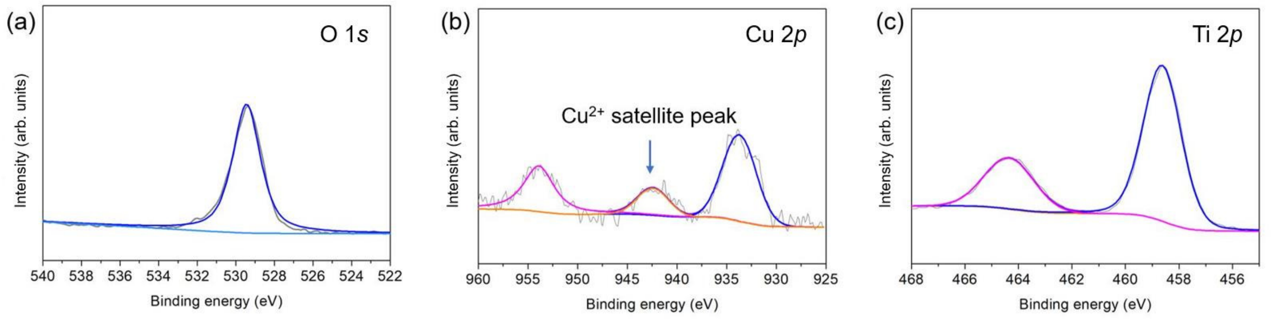

Supplementary Figure 6. (a) O 1*s*, (b) Cu 2*p*, and (c) Ti 2*p* XPS spectra of Cu-O/$Ti_{0.91}O_2$-SL.

Only one peak assigned to the lattice oxygen is detected in Cu-O/$Ti_{0.91}O_2$-SL, demonstrating V_O is absent in Cu-O/$Ti_{0.91}O_2$-SL. The g=2.003 signal of V_O is not detected in the EPR spectra of Cu-O/$Ti_{0.91}O_2$-SL (Fig. 2b). The EPR and O 1*s* XPS spectra confirm the absence of V_O in Cu-O/$Ti_{0.91}O_2$-SL.

The characteristic Cu^{2+} satellite peak in Cu 2*p* XPS spectrum and a distinct EPR Cu^{2+} signal at g= 2.043 (Fig. 2b) elucidate that Cu species is in a fully-oxidized state of +2 valance without obtaining electrons from the nearby Ti atoms in Cu-O/$Ti_{0.91}O_2$-SL.

Ti species in Cu-O/$Ti_{0.91}O_2$-SL remain almost unchanged compared with that in $Ti_{0.91}O_2$-SL, implying that Ti sites are almost unaffected by Cu single atoms when V_O is absent. Cu and Ti sites in Cu-O/$Ti_{0.91}O_2$-SL are in a relatively isolated form without detectable mutual electronic influence, which is consistent with the DFT results.

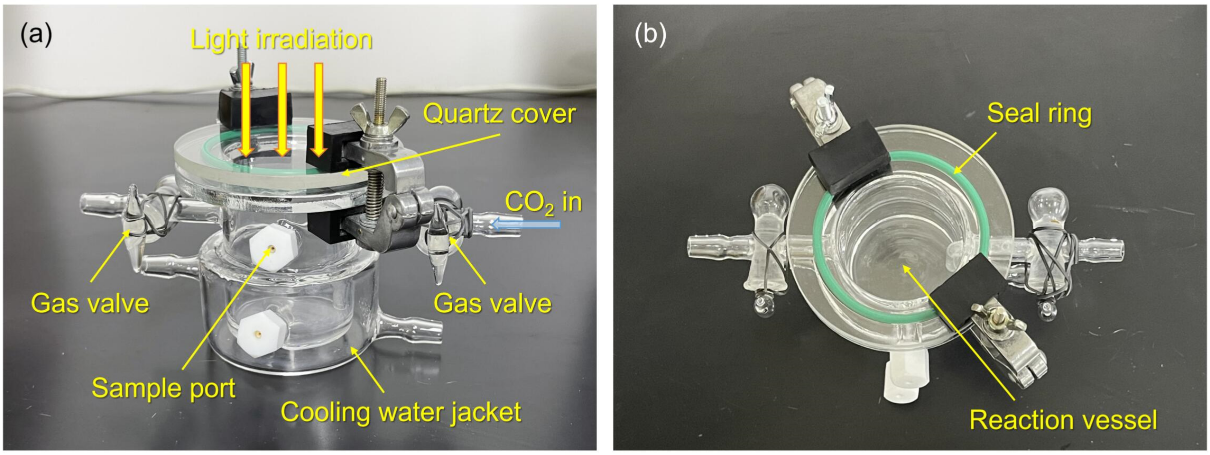

Supplementary Figure 7. (a) The side view and (b) top view of the photos for the reaction device of photocatalytic CO_2 reduction test.

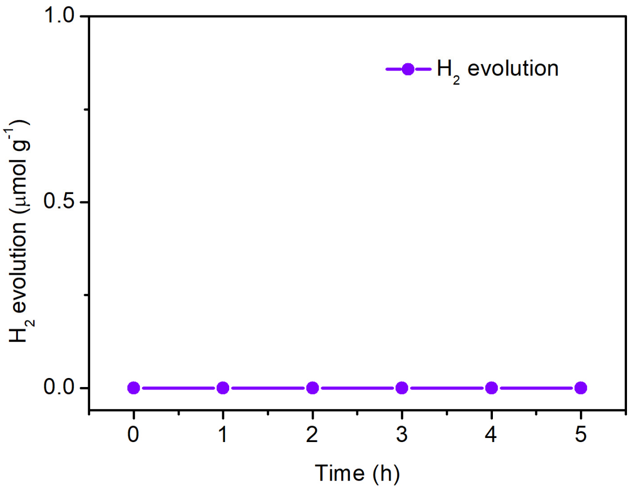

Supplementary Figure 8. H_2 evolution on Cu-Ti-V_O/$Ti_{0.91}O_2$-SL in acetonitrile aqueous solution.

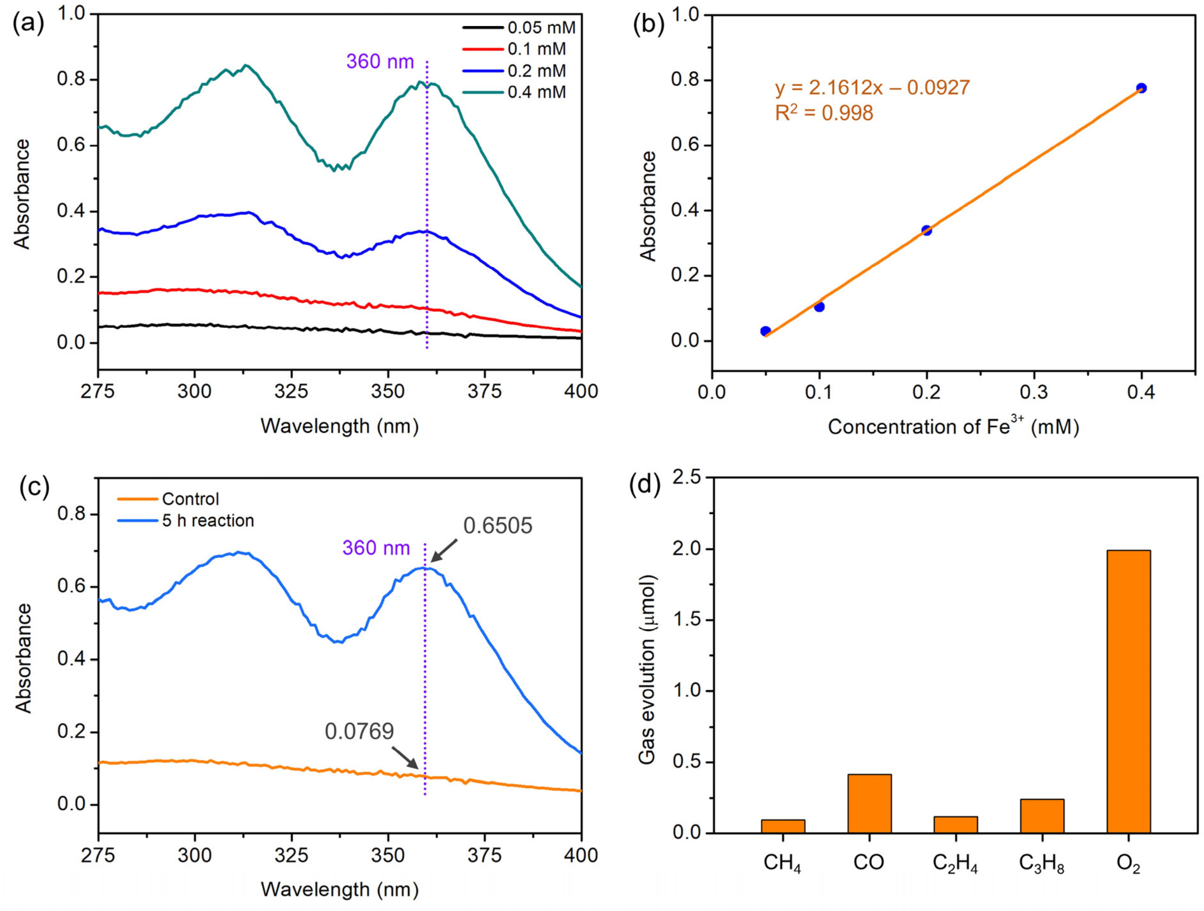

Supplementary Figure 9. (a) UV-vis absorption spectra of Fe^{III} at various concentrations. (b) The calibration curve of Fe^{III} obtained at 360 nm. (c) UV-vis absorption spectra of the control solution and the solution after 5 h photocatalytic reaction. (d) Generated products on Cu-Ti-V_O/$Ti_{0.91}O_2$-SL after 5 h reaction in acetonitrile aqueous solution.

To verify the production of O_2 in acetonitrile aqueous solution, we adopted the method reported by Zhang et al.[9]. The evolved O_2 was detected by adding Fe^{II} and quantifying its aerobic oxidation to Fe^{III} by UV−vis spectroscopy. After 5 h photocatalytic reaction, 100 μL of 150 mM aqueous Fe^{II} dissolved in 0.5 M aqueous HCl was added into the reaction solution (acetonitrile/water, 15 mL, v: v = 5:1), and the solution was diluted by 15 mL acetonitrile degassed by five pump-thaw cycles then

purged with Ar for 30 min. The absorbance of the diluted solution after 5 h reaction was determined by UV−vis spectroscopy (blue line in Supplementary Fig. 9c). The control spectrum was obtained by purging blank solution (acetonitrile/water, 15 mL, v: v = 5:1) with CO_2 for 1 h, adding 100 μL of 150 mM aqueous Fe^{II} dissolved in 0.5 M aqueous HCl, and diluted by 15 mL acetonitrile degassed by five pump-thaw cycles then purged with Ar for 30 min. The absorbance of the control solution was determined by UV−vis spectroscopy (orange line in Supplementary Fig. 9c). The difference of these two lines is due to the oxidation of Fe^{II} to Fe^{III} by the produced O_2 in 5 h catalytic reaction.

A calibration curve for the UV-vis absorption spectra of Fe^{III} at various concentrations was obtained at 360 nm (Supplementary Figs. 9a and b). The absorbance of the solution after 5 h reaction was measured 0.6505, and that of control solution was 0.0769, and the corresponding concentration of Fe^{III} was calculated 0.3439 and 0.0785 mM, respectively, according to the calibration curve. The calculation of O_2 production is shown as follows:

$$\mathbf{4}Fe^{2+} + O_2 + \mathbf{4}H^+ \rightarrow \mathbf{4}Fe^{3+} + \mathbf{2}H_2O$$

Amount of O_2 in the solution after catalytic reduction: 0.3439/4 mM × 30 mL = 2.5790 μmol.

Amount of O_2 in the control solution: 0.0785/4 mM × 30 mL = 0.5884 μmol.

Thus, the generated O_2 in the 5 h catalytic reaction is calculated to be 1.9906 μmol, equivalent to ~ 7.962 μmol of electrons. The reduction products in the 5 h catalytic reaction were measured by gas chromatography. ~ 0.416 μmol of CO, ~ 0.094 μmol of CH_4, ~ 0.118 μmol of C_2H_4, and ~ 0.242 μmol of C_3H_8 were generated after 5 h reaction, and the sum of equivalent electrons is ~ 7.84 μmol. The close match of the number of electrons participating in O_2 evolution and reduction half-reaction confirms O_2 was generated stoichiometrically as the oxidation products over Cu-Ti-V_O/$Ti_{0.91}O_2$-SL.

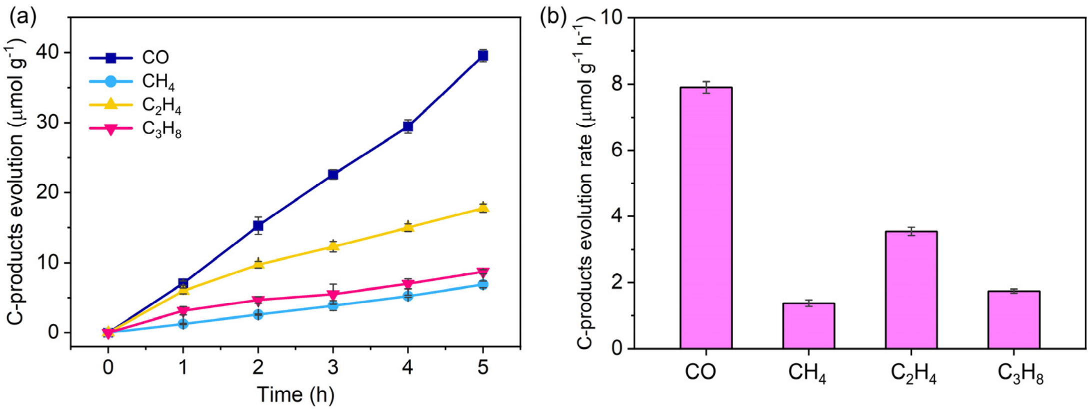

Supplementary Figure 10. (a) Photocatalytic carbon-based product evolution as a function of light irradiation time, and (b) carbon-based product formation rates on Cu-Ti-V_O/$Ti_{0.91}O_2$-SL in pure water. (Error bars indicate standard deviations.)

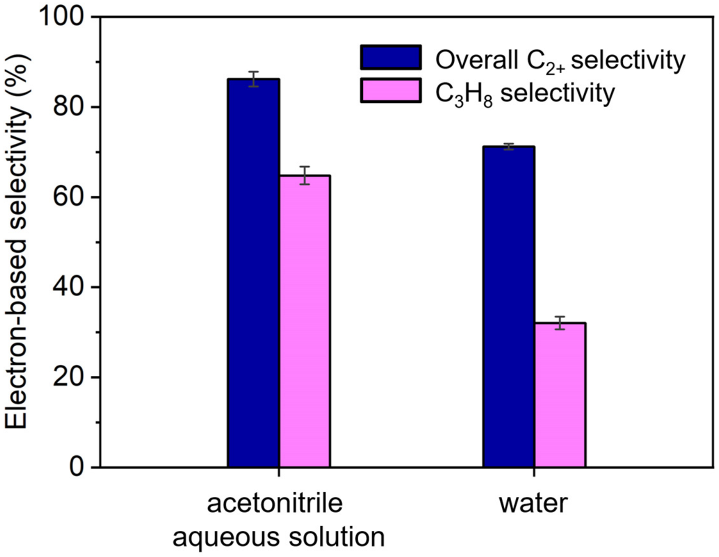

Supplementary Figure 11. Comparison for selectivity of total C_{2+} products and C_3H_8 over Cu-Ti-V_O/$Ti_{0.91}O_2$-SL in acetonitrile aqueous solution and pure water. (Error bars indicate standard deviations.)

H_2 is produced as side product with a formation rate of ~2.2 μmol g^{-1} h^{-1} in pure water.

The electron-based selectivity of C_3H_8 in pure water was calculated using:

$$\mathrm{Sel_{electron}}\ (C_3H_8) = \left(\frac{n\ (C_3H_8) \times 20}{n\ (CO)\times 2 + n\ (CH_4)\times 8 + n\ (C_2H_4)\times 12 + n\ (C_3H_8)\times 20 + n\ (H_2)\times 2}\right) \times 100\ \%$$

The electron-based selectivity of C_{2+} products in pure water was calculated using:

$$\mathrm{Sel_{electron}}\ (C_{2+}) = \left(\frac{n\ (C_2H_4)\times 12 + n\ (C_3H_8)\times 20}{n\ (CO)\times 2 + n\ (CH_4)\times 8 + n\ (C_2H_4)\times 12 + n\ (C_3H_8)\times 20 + n\ (H_2)\times 2}\right) \times 100\%$$

where *n* is the formation rate.

The electron-based selectivity of overall C_{2+} products over Cu-Ti-V_O/$Ti_{0.91}O_2$-SL in acetonitrile aqueous solution is 86.2% and that of C_3H_8 is 64.8%. Yet, the electron-based selectivity of overall C_{2+} products of Cu-Ti-V_O/$Ti_{0.91}O_2$-SL in pure water decreases to 71.2% and that of C_3H_8 decreases to 32.1%.

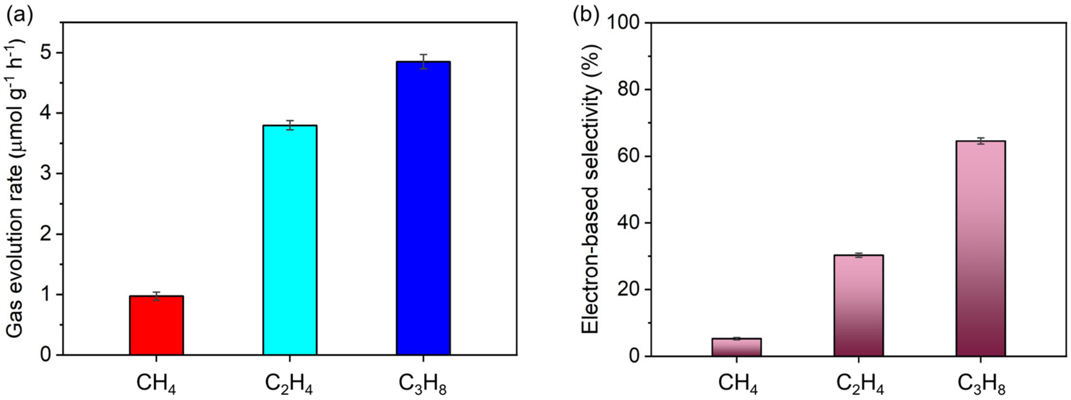

Supplementary Figure 12. (a) Formation rate and (b) electron-based selectivity of CH_4, C_2H_4 and C_3H_8 of photocatalytic CO reduction on Cu-Ti-V_O/$Ti_{0.91}O_2$-SL in acetonitrile aqueous solution. (Error bars indicate standard deviations.)

The electron-based selectivity of CH_4 was calculated using:

$$\mathrm{Sel}_{\mathrm{electron}}\ (\mathrm{CH_4}) = \left(\frac{n\ (\mathrm{CH_4})\times 8}{n\ (\mathrm{CH_4})\times 8 + n\ (\mathrm{C_2H_4})\times 12 + n\ (\mathrm{C_3H_8})\times 20}\right) \times 100\ \%$$

The electron-based selectivity of C_2H_4 was calculated using:

$$\mathrm{Sel}_{\mathrm{electron}}\ (\mathrm{C_2H_4}) = \left(\frac{n\ (\mathrm{C_2H_4})\times 12}{n\ (\mathrm{CH_4})\times 8 + n\ (\mathrm{C_2H_4})\times 12 + n\ (\mathrm{C_3H_8})\times 20}\right) \times 100\ \%$$

The electron-based selectivity of C_3H_8 was calculated using:

$$\mathrm{Sel}_{\mathrm{electron}}\ (\mathrm{C_3H_8}) = \left(\frac{n\ (\mathrm{C_3H_8})\times 20}{n\ (\mathrm{CH_4})\times 8 + n\ (\mathrm{C_2H_4})\times 12 + n\ (\mathrm{C_3H_8})\times 20}\right) \times 100\ \%$$

where n is the formation rate.

The electron-based selectivity of CH_4, C_2H_4 and C_3H_8 over Cu-Ti-V_O/$Ti_{0.91}O_2$-SL for CO reduction reaction in acetonitrile aqueous solution is 5.2%, 30.3% and 64.5%, respectively.

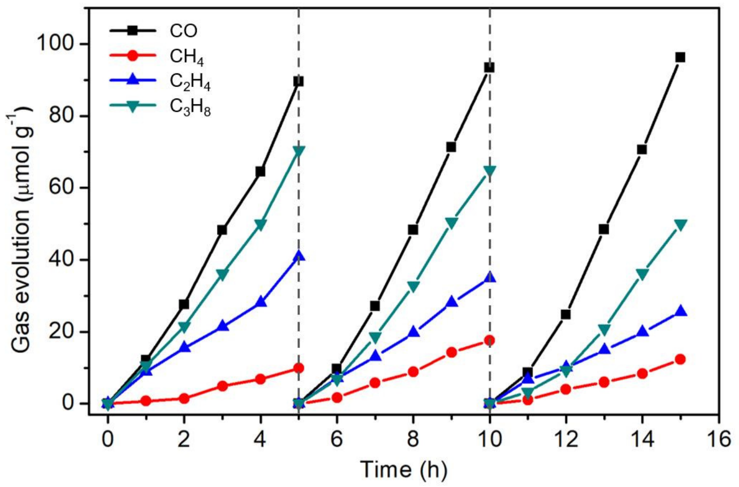

Supplementary Figure 13. Gas evolution amounts as a function of light irradiation time for Cu-Ti-V_O/$Ti_{0.91}O_2$-SL over 3 cycling tests in acetonitrile aqueous solution.

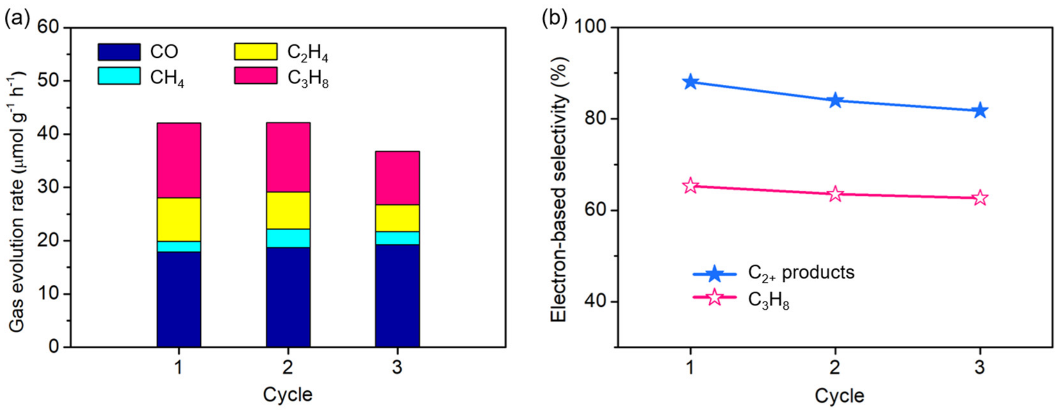

Supplementary Figure 14. (a) Product formation rate and (b) electron-based selectivity on Cu-Ti-$V_O/Ti_{0.91}O_2$-SL over 3 cycling tests in acetonitrile aqueous solution.

The electron-based selectivity of C_3H_8 was calculated using:

$$\mathrm{Sel_{electron}}\ (\mathrm{C_3H_8}) = \left(\frac{n\ (\mathrm{C_3H_8}) \times 20}{n\ (\mathrm{CO})\times 2 + n\ (\mathrm{CH_4}) \times 8 + n\ (\mathrm{C_2H_4})\times 12 + n\ (\mathrm{C_3H_8})\times 20}\right) \times 100\ \%$$

The electron-based selectivity of C_{2+} products was calculated using:

$$\mathrm{Sel_{electron}}\ (\mathrm{C_{2+}}) = \left(\frac{n\ (\mathrm{C_2H_4})\times 12 + n\ (\mathrm{C_3H_8})\times 20}{n\ (\mathrm{CO})\times 2 + n\ (\mathrm{CH_4})\times 8 + n\ (\mathrm{C_2H_4})\times 12 + n\ (\mathrm{C_3H_8})\times 20}\right) \times 100\%$$

where n is the formation rate.

The electron-based selectivity of C_{2+} products on Cu-Ti-$V_O/Ti_{0.91}O_2$-SL over 3 cycling tests in acetonitrile aqueous solution is 88.1%, 84.0% and 81.8%, respectively, and those of C_3H_8 is 65.3%, 63.5 and 62.7%, respectively.

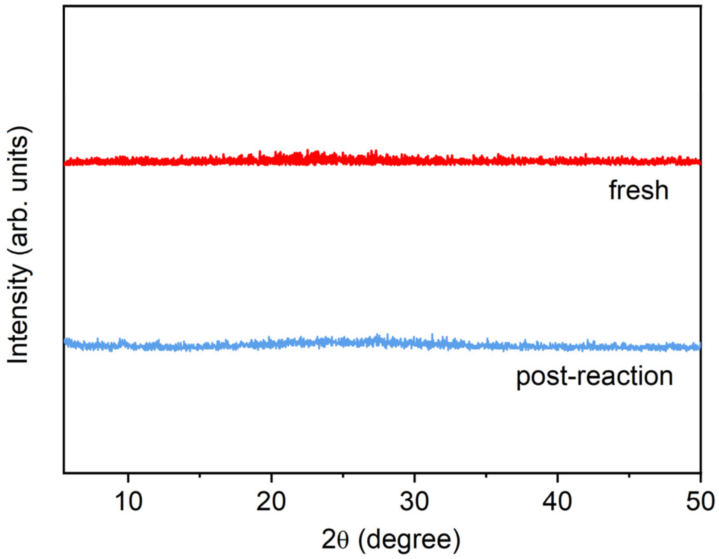

Supplementary Figure 15. XRD patterns for Cu-Ti-V_O/$Ti_{0.91}O_2$-SL before and after the photocatalytic CO_2 reduction reaction in acetonitrile aqueous solution.

The XRD patterns of the Cu-Ti-V_O/$Ti_{0.91}O_2$-SL after the reaction remained unchanged compared with that before the reaction, and no distinct Bragg reflection was observed, suggesting that no restacking occurs during the catalytic reaction.

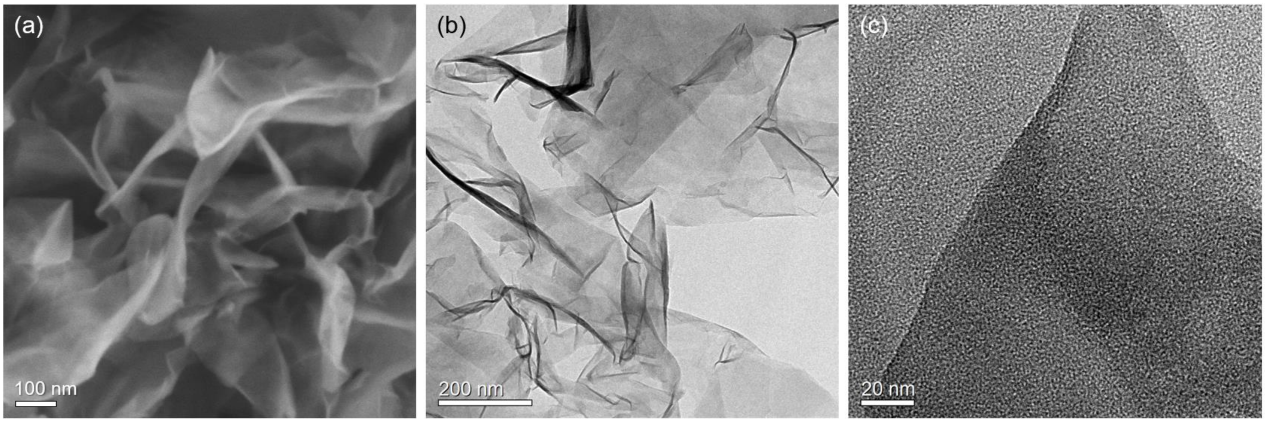

Supplementary Figure 16. (a) FE-SEM and (b, c) TEM images of Cu-Ti-V_O/$Ti_{0.91}O_2$-SL after the photocatalytic CO_2 reduction reaction.

The overall morphology of Cu-Ti-V_O/$Ti_{0.91}O_2$-SL remained unchanged after the reaction. Both FE-SEM and TEM images displayed the ultrathin sheet-like structure with a smooth surface, and no discernible nanoparticle was observed, implying the single-atom dispersion of Cu atoms after the catalytic test.

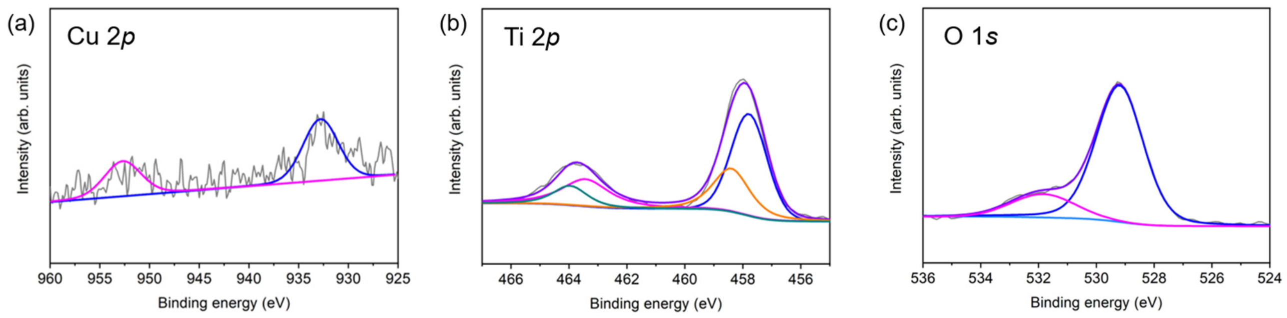

Supplementary Figure 17. (a) Cu 2*p*, (b) Ti 2*p*, and (c) O 1*s* XPS spectra of Cu-Ti-V_O/$Ti_{0.91}O_2$-SL after the photocatalytic CO_2 reduction reaction.

The XPS results for the Cu-Ti-V_O/$Ti_{0.91}O_2$-SL catalyst after the photocatalytic CO_2 reduction reaction reveal that the chemical states of Cu, Ti, and O did not undergo significant changes during the reaction.

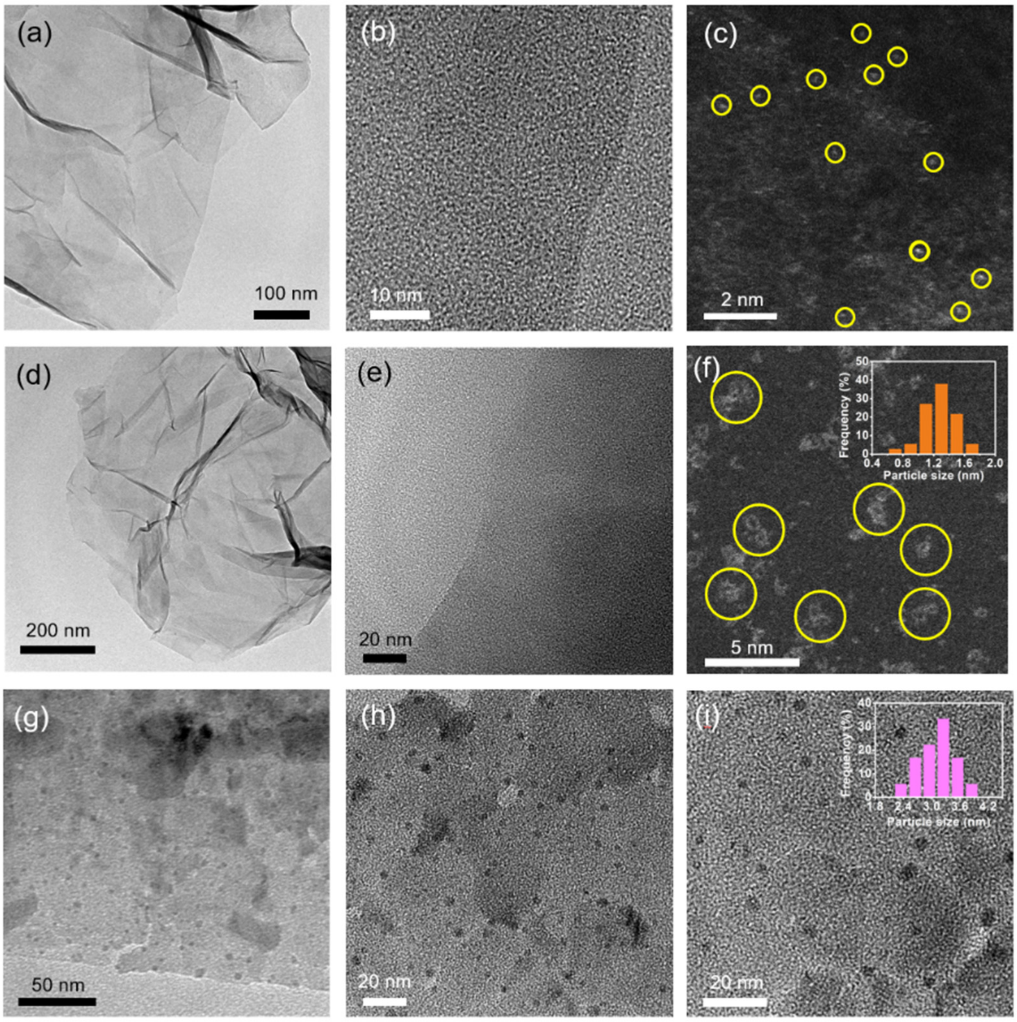

Supplementary Figure 18. (a, b) TEM, (c) AC HAADF-STEM images of Cu-Ti-V_O/$Ti_{0.91}O_2$-SL(lower). (d, e) TEM, (f) AC HAADF-STEM images of Cu NC/$Ti_{0.91}O_2$-SL. (g-i) TEM images of Cu NP/$Ti_{0.91}O_2$-SL. The insets in (f) and (i) are the particle size distributions of Cu NC and NP, respectively.

With decrease of Cu loading amount, Cu sites remain atomically dispersed but with a sparser distribution (denoted as Cu-Ti-V_O/$Ti_{0.91}O_2$-SL(lower), Cu loading mass of 0.10 wt%). Cu nanoclusters with an average size of ~ 1.27 nm emerge when the Cu loading mass increases to 2.11 wt% (denoted as Cu NC/$Ti_{0.91}O_2$-SL). Higher Cu loading of 3.70 wt%, together with longer RTT time, leads to Cu nanoparticles with an average size of ~ 3.06 nm (denoted as Cu NP/$Ti_{0.91}O_2$-SL).

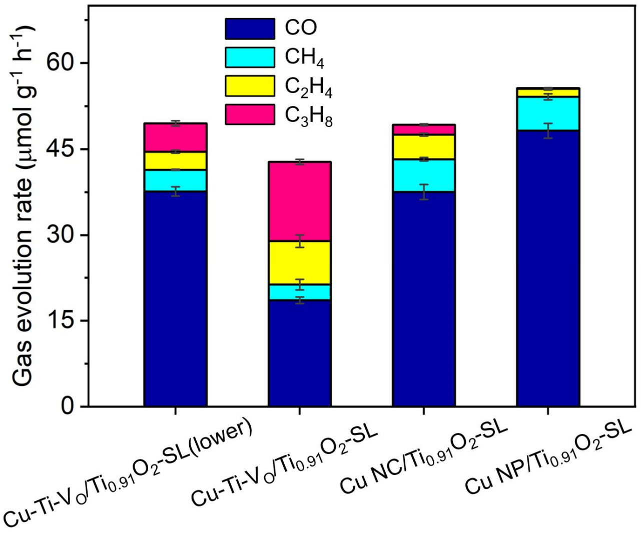

Supplementary Figure 19. Comparison of the product formation rates on Cu-Ti-V_O/$Ti_{0.91}O_2$-SL and Cu-Ti-V_O/$Ti_{0.91}O_2$-SL (lower), and Cu NC/$Ti_{0.91}O_2$-SL, and Cu NP/$Ti_{0.91}O_2$-SL in acetonitrile aqueous solution. (Error bars indicate standard deviations.)

The electron-based selectivity of C_{2+} products was calculated using:

$$\mathrm{Sel}_{\mathrm{electron}}\ (\mathrm{C}_{2+}) = \left(\frac{n\ (\mathrm{C_2H_4})\times 12 + n\ (\mathrm{C_3H_8})\times 20}{n\ (\mathrm{CO})\times 2 + n\ (\mathrm{CH_4})\times 8 + n\ (\mathrm{C_2H_4})\times 12 + n\ (\mathrm{C_3H_8})\times 20}\right) \times 100\%$$

where n is the formation rate.

The electron-based selectivity of C_{2+} products on Cu-Ti-V_O/$Ti_{0.91}O_2$-SL (lower), and Cu NC/$Ti_{0.91}O_2$-SL, and Cu NP/$Ti_{0.91}O_2$-SL is 56.5%, 41.5% and 11.7%. The inferior C_{2+} selectivity of Cu NC/$Ti_{0.91}O_2$-SL and Cu NP/$Ti_{0.91}O_2$-SL is probably because that Cu-Ti-V_O units related to C-C coupling cannot effectively form between Ti atoms and Cu NCs or NPs. The interaction between metal and the oxide matrix become weaker as the metal particle size get larger, and when the particle size got large enough (>2 nm), the metal-oxide interaction is too weak to influence the catalytic properties[10,11].

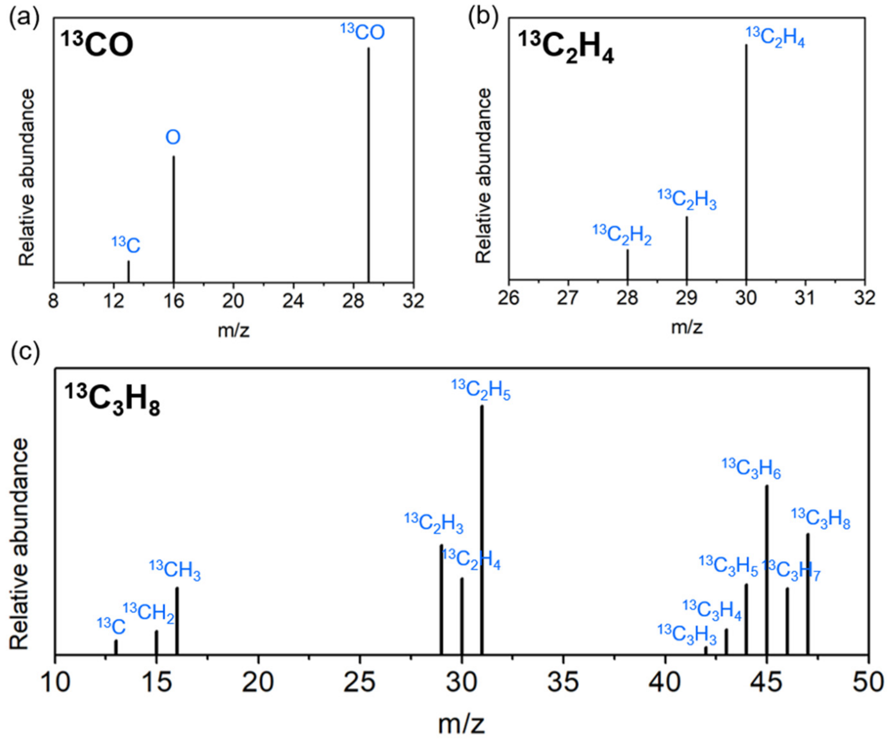

Supplementary Figure 20. Mass spectra of (a) CO, (b) C_2H_4, and (c) C_3H_8 when the input gas was $^{13}CO_2$ for photocatalytic CO_2 reduction on Cu-Ti-V_O/$Ti_{0.91}O_2$-SL.

Isotropic experiment using $^{13}CO_2$ as the feedstock gas was performed to investigate the carbon source of the products. The MS peak at m/z = 47 is the base peak of $^{13}C_3H_8$, and the fragment peaks at m/z = 46, 45, 44, 43, 42, 31, 30, 29, 16, 15 and 13 are attributed to $^{13}C_3H_7$, $^{13}C_3H_6$, $^{13}C_3H_5$, $^{13}C_3H_4$, $^{13}C_3H_3$, $^{13}C_2H_5$, $^{13}C_2H_4$, $^{13}C_2H_3$, $^{13}CH_3$, $^{13}CH_2$ and ^{13}C, respectively. The MS peak at m/z = 30 is the base peak of $^{13}C_2H_4$, and the fragment peaks at m/z = 29 and 28 are assigned to $^{13}C_2H_3$ and $^{13}C_2H_2$, respectively. The MS peak at m/z = 29 is the base peak of ^{13}CO, and the fragment peaks at m/z = 16 and 13 are ascribed to O and ^{13}C, respectively. The above results unambiguously confirm that the carbon source of the carbon products originates from the input CO_2.

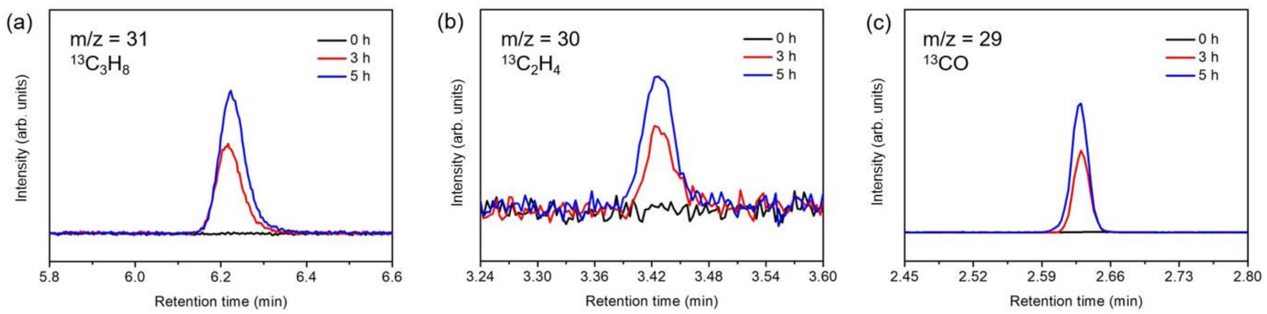

Supplementary Figure 21. Mass chromatography spectra of (a) $^{13}C_3H_8$ (m/z =31), (b) $^{13}C_2H_4$ (m/z =30), and (c) ^{13}CO (m/z =29) generated on Cu-Ti-V_O/$Ti_{0.91}O_2$-SL under $^{13}CO_2$ atmosphere at different irradiation time.

The isotopic analysis results display ever-increasing mass spectra peaks of $^{13}C_3H_8$ (m/z = 31), $^{13}C_2H_4$ (m/z = 30) and ^{13}CO (m/z = 29). The formation rate of $^{13}C_3H_8$ is calculated ~13.2 μmol g^{-1} h^{-1}, which matches well with the results of $^{12}CO_2$.

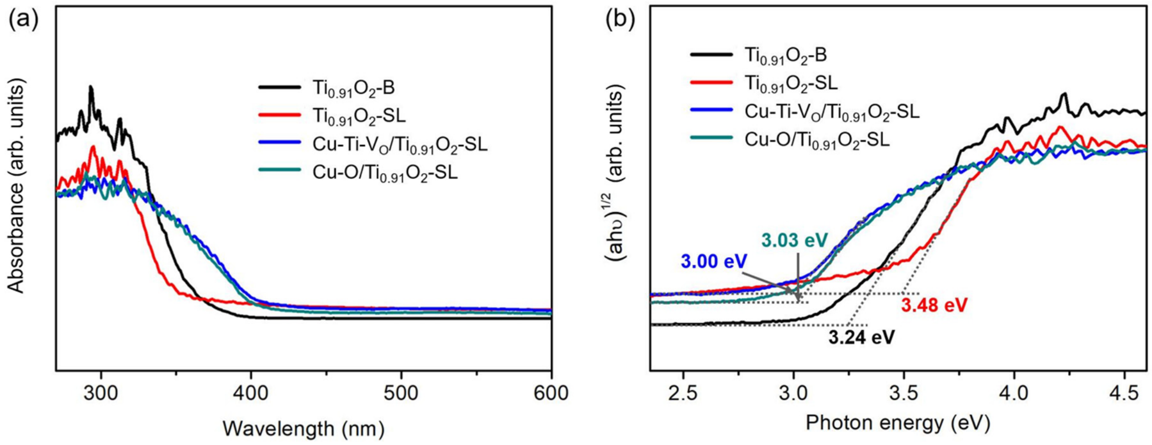

Supplementary Figure 22. (a) UV−vis DRS and (b) corresponding band edges of $Ti_{0.91}O_2$-B, $Ti_{0.91}O_2$-SL, Cu-O/$Ti_{0.91}O_2$-SL, and Cu-Ti-V_O/$Ti_{0.91}O_2$-SL.

The absorption edge of single-layer $Ti_{0.91}O_2$-SL displays a blue shift compared with layered $Ti_{0.91}O_2$-B due to the quantum confinement effect of the monolayer structure, and the bandgap of $Ti_{0.91}O_2$-SL and $Ti_{0.91}O_2$-B is calculated 3.48 and 3.24 eV, respectively. The light absorption is enhanced with the implantation of Cu single atoms. Cu-Ti-V_O/$Ti_{0.91}O_2$-SL and Cu-O/$Ti_{0.91}O_2$-SL exhibit similar absorption edge, with the bandgap of 3.00 and 3.03 eV, respectively, indicating better light utilization than pure $Ti_{0.91}O_2$-SL.

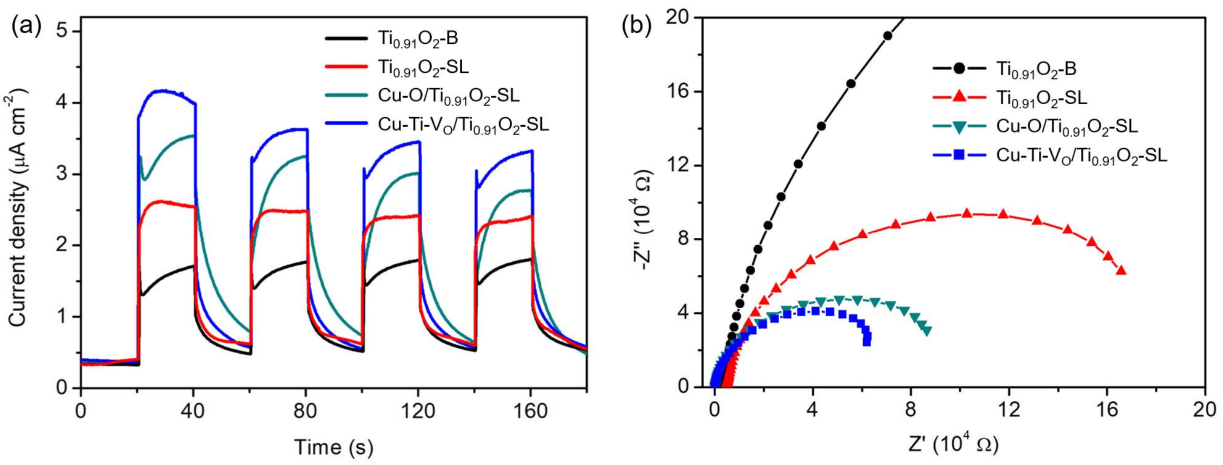

Supplementary Figure 23. (a) Photocurrent response, and (b) Nyquist plots of $Ti_{0.91}O_2$-B, $Ti_{0.91}O_2$-SL, Cu-O/$Ti_{0.91}O_2$-SL, and Cu-Ti-V_O/$Ti_{0.91}O_2$-SL.

The separation and migration of photogenerated charge carriers are investigated by photoelectrochemical (PEC) analysis. Single-layer $Ti_{0.91}O_2$-SL affords higher photocurrent density than layered $Ti_{0.91}O_2$-B, ascribed to the promoted charge separation and migration of the 2D atomically-thin structure, which effectively shortens the charge transfer distance from body to surface, and largely decreases charge recombination possibility. Cu-Ti-V_O/$Ti_{0.91}O_2$-SL exhibits higher photocurrent density than pure $Ti_{0.91}O_2$-SL, indicating further enhancement of charge separation and transfer efficiency after Cu introduction. Also, electrochemical impedance spectra (EIS) reveal that Cu-Ti-V_O/$Ti_{0.91}O_2$-SL manifests the smallest semicircle in Nyquist plots, suggesting the lowest charge-transfer resistance, which allows for fast transport of photoinduced charge carriers. The above results demonstrate that the Cu-Ti-V_O/$Ti_{0.91}O_2$-SL exhibits fast charge carrier kinetics, favorable to the multi-electron reactions of generating C_{2+} products.

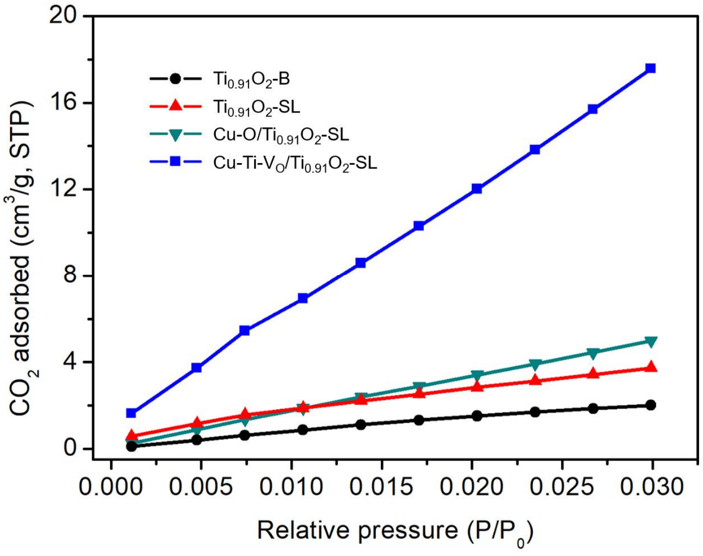

Supplementary Figure 24. CO_2 adsorption isotherms at 273 K of $Ti_{0.91}O_2$-B, $Ti_{0.91}O_2$-SL, Cu-O/$Ti_{0.91}O_2$-SL, and Cu-Ti-V_O/$Ti_{0.91}O_2$-SL.

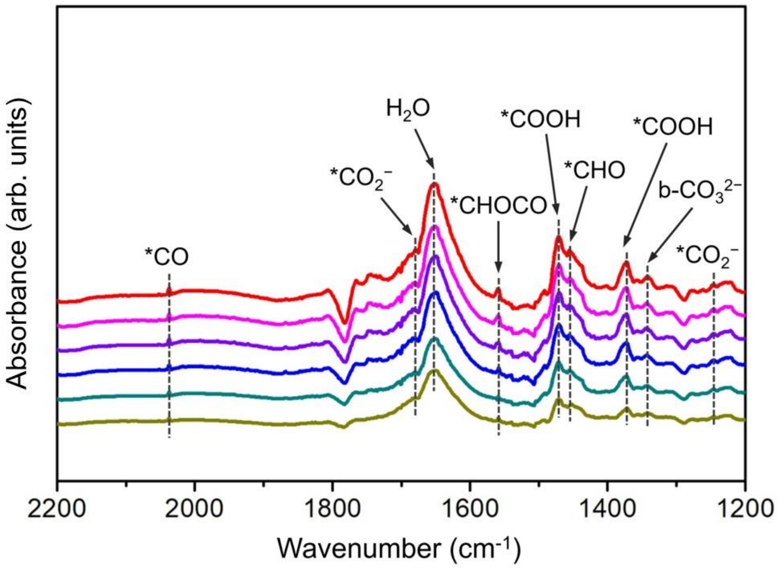

Supplementary Figure 25. In situ DRIFTS spectra of the photocatalytic reduction of $^{13}CO_2$ on Cu-Ti-V_O/$Ti_{0.91}O_2$-SL.

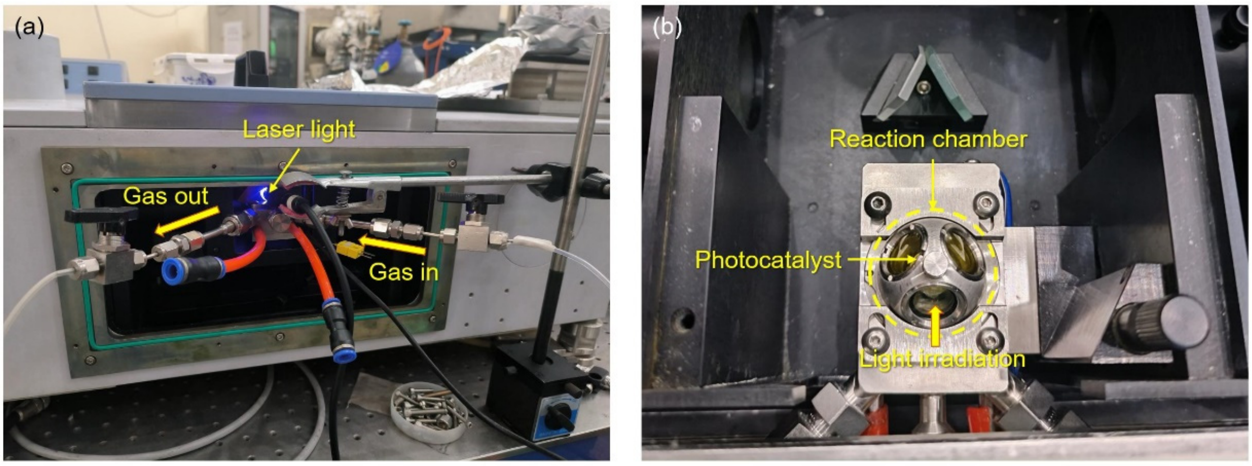

Supplementary Figure 26. (a) The side view and (b) top view of the photos for the instrument of in situ DRIFTS measurements.

The photocatalyst was placed inside a gas-tight chamber with transparent windows which allow for the light irradiation from the side-on. The 365 nm laser light was used to initiate the CO_2 photoreduction reaction inside the chamber. The sample was first swept with Ar gas to purge out surface adsorbates. Then CO_2 and water vapor were introduced into the chamber. Under light irradiation, the DRIFTS spectra were obtained every 5 minutes at room temperature.

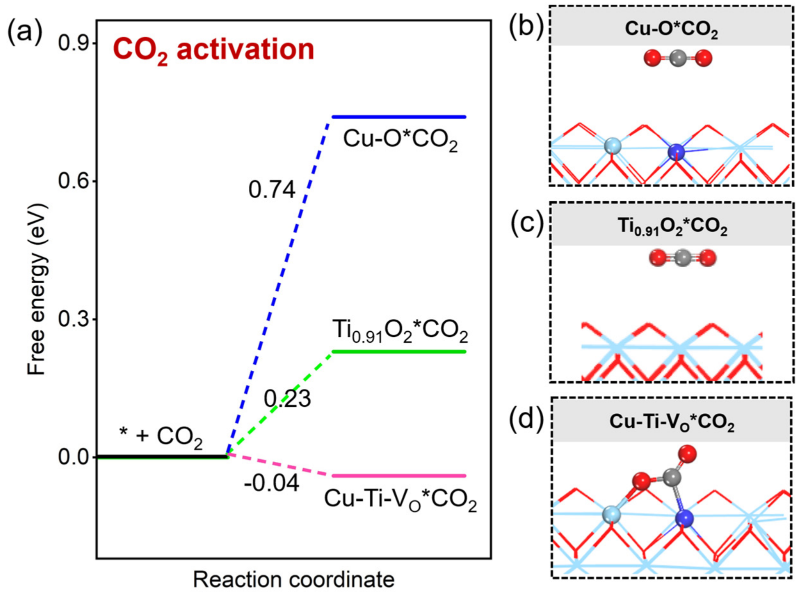

Supplementary Figure 27. (a) The Gibbs free energy changes of CO_2 activation, and the corresponding adsorption configurations of $*CO_2$ on (b) Cu-O site, (c) $Ti_{0.91}O_2$ matrix, and (d) Cu-Ti-V_O unit (Colour codes in the atomic models: light blue (Ti), blue (Cu), red (O) and grey (C)).

The CO_2 activation on different reaction centers were studied by DFT methods. On Cu-Ti-V_O unit. The absorbed CO_2 is considered to be efficiently activated to a bent configuration with an angle of 130.6°, and the Gibbs free energy change is -0.04 eV. Yet, on Cu-O site and $Ti_{0.91}O_2$ domain, CO_2 molecule retains a nearly linear geometry with uphill energy changes, indicating the sluggish CO_2 reduction.

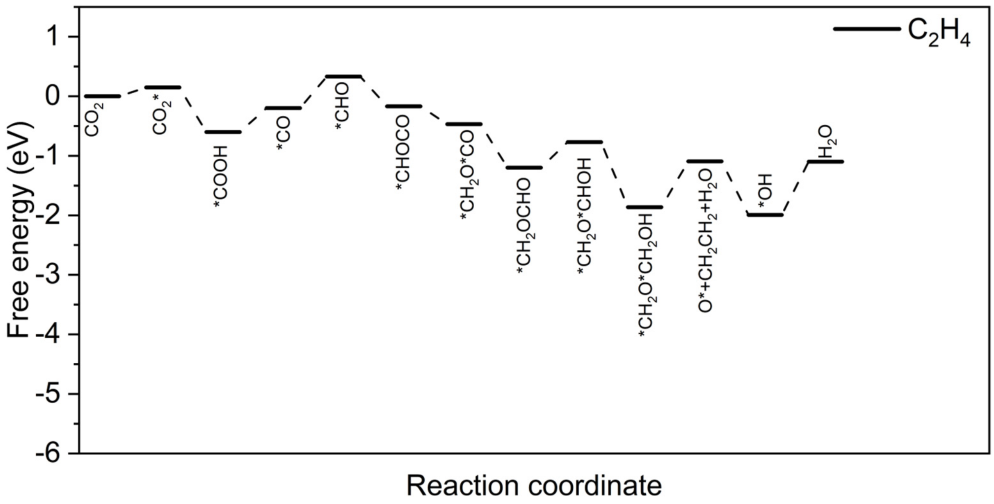

Supplementary Figure 28. Gibbs free energy diagrams of CO_2 reduction to C_2H_4 on Cu-Ti-V_O units.

The potential determining step (PDS) of ethylene pathway is the hydrogenation of *OH with a free energy change of 0.9 eV, and the PDS of propane pathway is the protonation of $^*CH_2OCOHCH_2$ to $^*CH_2OHCOHCH_2$ with a free energy change of 0.62 eV.

$CO_2 + * \rightarrow *CO_2$			(1)
$*CO_2 + H^+ + e^- \rightarrow *COOH$			(2)
$*COOH + e^- + H^+ \rightarrow *CO + H_2O$			(3)
$*CO + e^- + H^+ \rightarrow *CHO$	or	$*CO \rightarrow$ **$CO\uparrow$** $+ *$	(4)
$*CHO + CO \rightarrow *CHOCO$			(5)
$*CHOCO + e^- + H^+ \rightarrow *CH_2OCO$			(6)
$*CH_2OCO + CO \rightarrow *CH_2OCOCO$		$*CH_2OCO + e^- + H^+ \rightarrow *CH_2OCHO$	(7)
$*CH_2OCOCO + e^- + H^+ \rightarrow *CH_2OCOCHO$		$*CH_2OCHO + e^- + H^+ \rightarrow *CH_2OCHOH$	(8)
$*CH_2OCOCHO + e^- + H^+ \rightarrow *CH_2OCOCHOH$	or	$*CH_2OCHOH + e^- + H^+ \rightarrow *CH_2OCH_2OH$	(9)
$*CH_2OCOCHOH + e^- + H^+ \rightarrow *CH_2OCOCH_2OH$		$*CH_2OCH_2OH + e^- + H^+ \rightarrow *O +$ **$C_2H_4\uparrow$** $+ H_2O$	(10)
$*CH_2OCOCH_2OH + e^- + H^+ \rightarrow *CH_2OCOHCH_2OH$		$*O + e^- + H^+ \rightarrow *OH$	(11)
$*CH_2OCOHCH_2OH + e^- + H^+ \rightarrow *CH_2OCOHCH_2 + H_2O$		$*OH + e^- + H^+ \rightarrow H_2O + *$	(12)
$*CH_2OCOHCH_2 + e^- + H^+ \rightarrow *CH_2OHCOHCH_2$			(13)
$*CH_2OHCOHCH_2 + e^- + H^+ \rightarrow *CH_2OHCHOHCH_2$			(14)
$*CH_2OHCHOHCH_2 + e^- + H^+ \rightarrow *CH_2OHCHCH_2 + H_2O$			(15)
$*CH_2OHCHCH_2 + e^- + H^+ \rightarrow *CH_2CHCH_2 + H_2O$			(16)
$*CH_2CHCH_2 + e^- + H^+ \rightarrow *CH_3CHCH_2$			(17)
$*CH_3CHCH_2 + e^- + H^+ \rightarrow *CH_3CH_2CH_2$			(18)
$*CH_3CH_2CH_2 + e^- + H^+ \rightarrow$ **$C_3H_8\uparrow$** $+ *$			(19)

Supplementary Figure 29. Possible reaction pathway for reduction of CO_2 into C_2H_4 and C_3H_8 on Cu-Ti-V_O/$Ti_{0.91}O_2$-SL under light illumination.

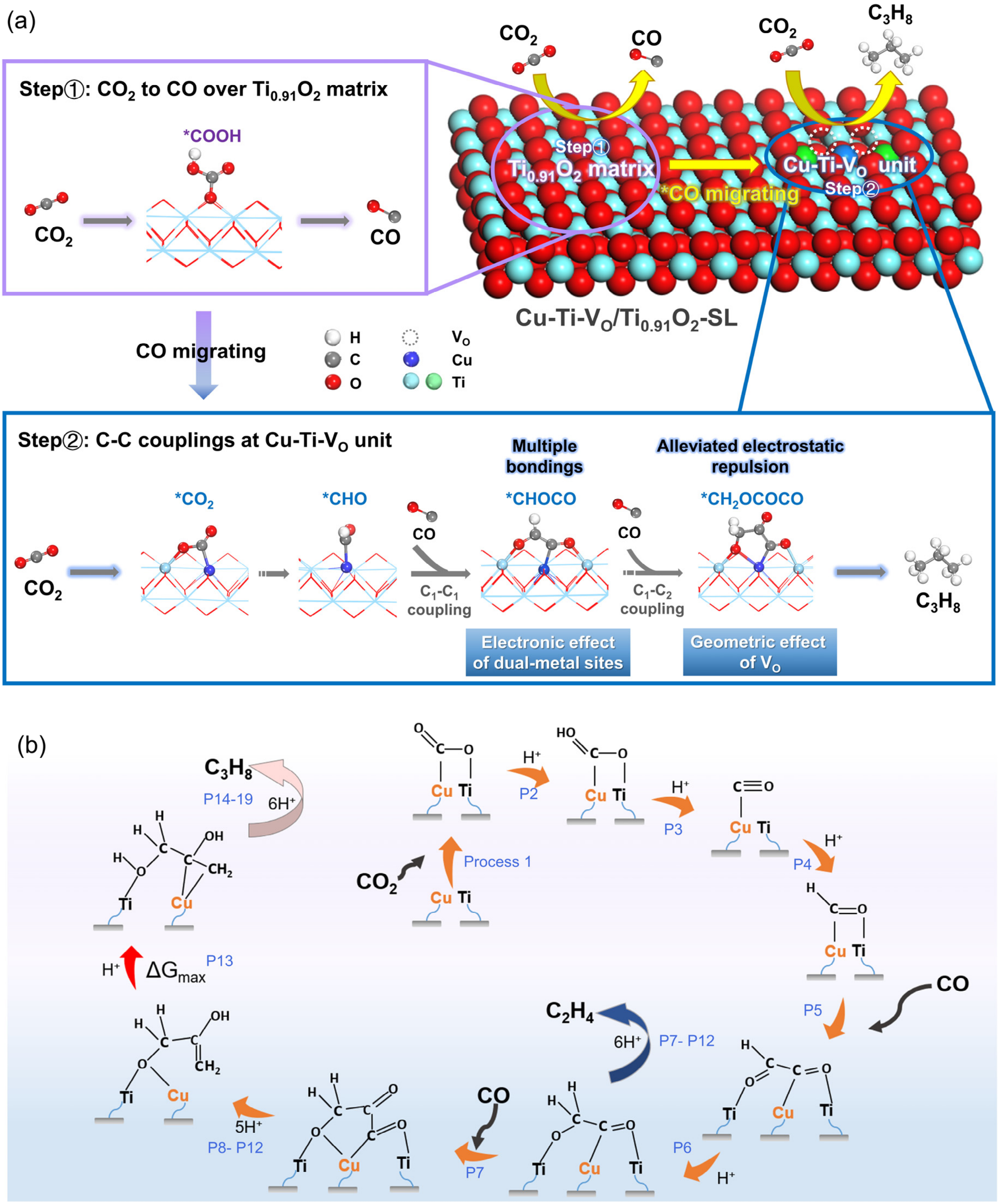

Supplementary Figure 30. (a) Illustration of the roles of the Cu atom-free $Ti_{0.91}O_2$ domain and Cu-Ti-V_O unit domain in tandem catalytic reduction of CO_2 to C_3H_8 on Cu-Ti-V_O/$Ti_{0.91}O_2$-SL. (b) Schematic pathways of C_{2+} hydrocarbon formation on Cu-Ti-V_O units.

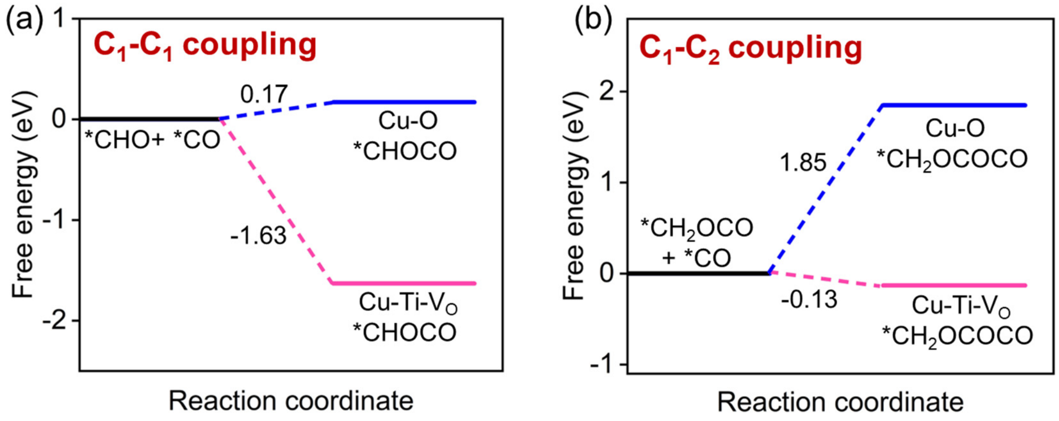

Supplementary Figure 31. Comparison for the Gibbs free energy changes of (a) C_1-C_1 coupling and (b) C_1-C_2 coupling on Cu-Ti-V_O unit and Cu-O site.

The Gibbs free energy changes of C_1-C_1 coupling (*CHO+ *CO→ *CHOCO) and C_1-C_2 coupling (*CH_2OCO+ *CO→ *CH_2OCOCO) on Cu-Ti-V_O unit and Cu-O site are investigated and summarized here. In these two C-C coupling reactions, the free energy levels of the resulting *CHOCO and *CH_2OCOCO intermediates are both lower than their corresponding starting *CHO and *CH_2OCO intermediates. However, the case is on the contrary for Cu-O sites, where C-C couplings are challenging endergonic process. Thus, it is deduced that the relatively low adsorption energy levels of *CHOCO and *CH_2OCOCO on Cu-Ti-V_O unit play a key role in determining the downhill energy changes of C-C coupling processes.

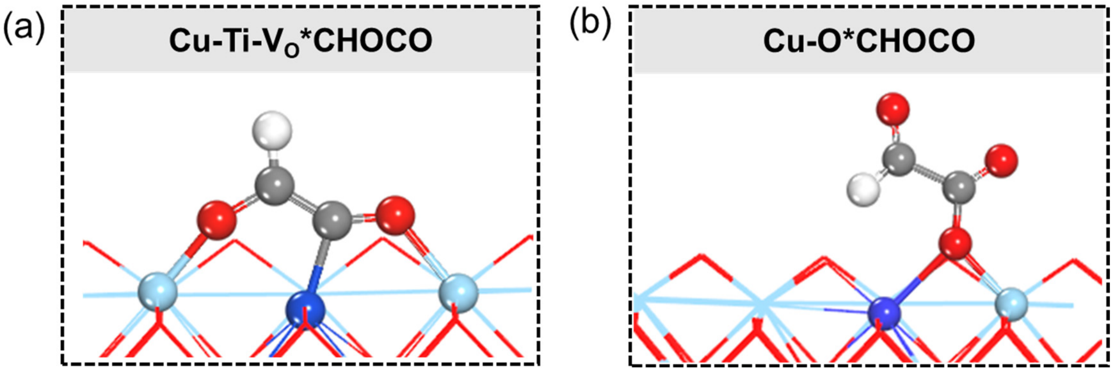

Supplementary Figure 32. Optimized adsorption configurations of *CHOCO on (a) Cu-Ti-V_O unit and (b) Cu-O site (Colour codes in the atomic models: light blue (Ti), blue (Cu), red (O), grey (C) and white (H)).

On Cu-Ti-V_O unit, both Cu and Ti sites are believed to be involved in the adsorption of *CHOCO. With an asymmetric electron density distribution, the electron-rich Cu centre may show affinity to the electron-poor C atom, and the electron-poor Ti centre tends to bond with the electron-rich O atoms. A multiple bonding configuration containing one Cu-C bond and two Ti-O bonds is subsequently built at the Cu-Ti-V_O unit, and 0.43 e^- are transferred from the Cu-Ti-V_O unit to *CHOCO, significantly strengthening the adsorption of *CHOCO and thus lowering its energy level. On Cu-O sites, only one C atom in *CHOCO bonds to the Cu-O site through a sole C-O bond. The configuration with single bonding is not strong enough, leading to an unstable adsorption mode and thus a high energy level of *CHOCO at Cu-O sites. Thus, it is deduced that the electronic effect of the dual-metal sites in Cu-Ti-V_O unit can effectively stabilize the *CHOCO intermediate to promote C_1-C_1 coupling.

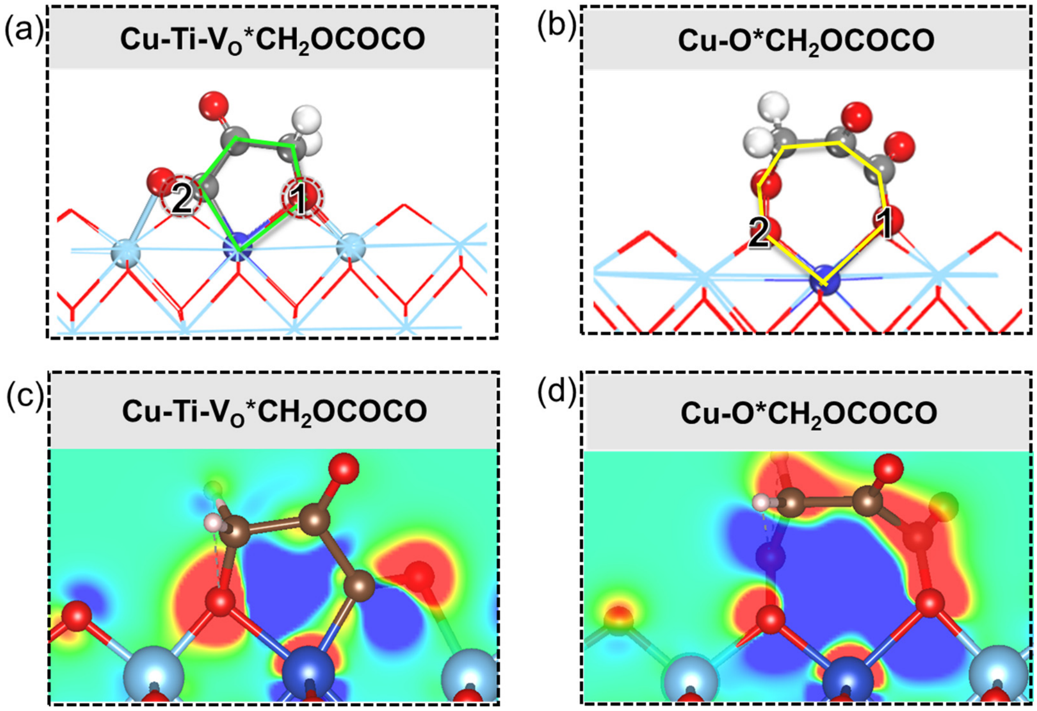

Supplementary Figure 33. Optimized adsorption configurations of $*CH_2OCOCO$ on (a) Cu-Ti-V_O unit and (b) Cu-O site. Charge density differences of $*CH_2OCOCO$ on (c) Cu-Ti-V_O unit and (d) Cu-O site (Colour codes in the atomic models: light blue (Ti), blue (Cu), red (O), grey (C), and white (H)).

For the relatively large-size $*CH_2OCOCO$ intermediate that contains multiple C and O atoms, the intermolecular and intramolecular electrostatic repulsion generally has a huge influence on the adsorption and its free energy level. When absorbed on Cu-Ti-V_O unit, $*CH_2OCOCO$ would occupy the place of V_O (position 1 in Supplementary Fig. 32a), forming a stable five-membered ring configuration consisting of one Cu atom, three C atoms and one O atom (highlighted in green lines in Supplementary Fig. 32a). Benefiting from the Vos, negligible distinct electron accumulation is expected on Cu-Ti-V_O unit, according to charge density differences results (Supplementary Fig. 32c), which may avoid the electrostatic repulsion and stabilize the adsorption of $*CH_2OCOCO$ to a lower energy level. Yet, when Vos are absent (position 1 and 2 in Supplementary Fig. 32b), $*CH_2OCOCO$

on Cu-O site tends to take a seven-membered ring configuration composed of one Cu atom, three C atoms and three O atoms (highlighted in yellow lines in Supplementary Fig. 32b). Dense electron accumulations and strong electrostatic repulsion are thereby presented, leading to the highly unstable adsorption and a very high energy level. Hence, it could be deduced that the geometric effect of Vos in Cu-Ti-V_O unit may contribute to the lowered adsorption energy level of *CH_2OCOCO to promoting C_1-C_2 coupling.

Supplementary Table 1. EXAFS fitting parameters at the Cu K-edge for Cu-Ti-V_O/$Ti_{0.91}O_2$-SL.

Shell	CN [a]	R (Å) [b]	σ^2 (Å^2·10^{-3}) [c]	ΔE_0 (eV) [d]	*R* factor (%)
Cu-O	3.6	1.93	4.4	-1.7	0.4
Cu-Ti	0.6	2.90	6.4	-9.6	

[a] *CN*: coordination numbers; [b] *R*: bond distance; [c] σ^2: Debye-Waller factors; [d] ΔE_0: the inner potential correction. *R* factor: goodness of fit. *S*02 were set as 0.84 for Cu-O, which were obtained from the experimental EXAFS fit of reference CuO by fixing CN as the known crystallographic value and was fixed to all the samples.

Supplementary Table 2. Optimized structural parameters using DFT for Cu-Ti-V_O/$Ti_{0.91}O_2$-SL.

**Shell**	**CN** [a]	**R (Å)** [b]
Cu-O	4	1.92
Cu-Ti	1	3.04

[a] *CN*: coordination numbers; [b] *R*: bond distance

Our calculation performed from the perfect TiO_2-SL, the calculated Ti-O bonding length is 1.98 Å, 2.20 Å, 1.84 Å, which is relatively 1.96 Å, 2.22 Å, 1.82 Å in previous reported lepidocrocite-like structure of TiO_2[12]. When introducing the Cu-Ti-V_O unit in the perfect TiO_2-SL through Cu SAs adsorption and two oxygen atoms vacancies, the Cu-O bonding length is 1.92 Å containing error from experimental result <1%. The Cu-Ti bonding length is 3.04 Å, being consistent with the experimental result (2.90 Å). In addition, a theoretical coordination number of 4 assigned to Cu-O coordination agrees with the EXAFS fitting analysis.

Supplementary Table 3. Performance comparison of reported photocatalytic CO_2 reduction to C_3 and C_2 products in non-acetonitrile and acetonitrile medium.

Photocatalyst	Reaction medium	Carbon-based products yield	C_{2+} products selectivity (%)	Ref.
Cu-Ti-V_O /$Ti_{0.91}O_2$-SL	CO_2-saturated acetonitrile aqueous solution	C_3H_8: 13.8 μmol g^{-1} h^{-1}<br>C_2H_4: 7.6 μmol g^{-1} h^{-1}<br>CO: 18.6 μmol g^{-1} h^{-1}	64.8 [a] /32.4 [b]<br>21.4 [a] /17.8 [b]	**This work**
Co_3O_4 molecular complexes on TiO_2/FTO	CO_2-saturated wet acetonitrile	CO: 923.1 μmol g^{-1} h^{-1}<br>CH_4: 294.3 μmol g^{-1} h^{-1}		13
$CsPbBr_3$ QDs /NH_x-rich g-C_3N_4	CO_2-saturated wet acetonitrile	CO: 149 μmol g^{-1} h^{-1}		14
MOF-808-CuNi	CO_2-saturated acetonitrile aqueous solution	CH_4: 158.7 μmol g^{-1} h^{-1}<br>CO: 2.3 μmol g^{-1} h^{-1}		15
Ni−Co_3O_4	Simulated air in acetonitrile aqueous solution	CO: 133.6 μmol (3 h)		16
BP QDs-$ZnIn_2S_4$	CO_2-saturated wet acetonitrile	CO: 15 μmol (4 h)		17
Mn SA on N-C	CO_2-saturated acetonitrile aqueous solution	CO: 1470 μmol g^{-1} h^{-1}		18
Au nanoparticles	CO_2-saturated EMIM-BF_4 aqueous solution	C_2H_4: N/A<br>C_2H_2: N/A<br>C_3H_6: N/A<br>C_3H_8: N/A<br>CH_4: N/A	~ 16 [b]<br>~ 12 [b]<br>~ 10 [b]<br>~ 6 [b]	19
0.02%Co-1%Cu/TiO_2	CO_2/water vapor	C_2H_6: 267.60 μmol (3 h)<br>C_3H_8: 10.07 μmol (3 h)<br>CH_4: 169.79 μmol (3 h)<br>CO: 150.58 μmol (3 h)	~ 44.7 [b]<br>~1.7 [b]	20
CuOx@p-ZnO	CO_2/water vapor	C_2H_4: 2.7 μmol g^{-1} h^{-1}	32 [b]	21

		CO: 3.3 μmol g^{-1} h^{-1}<br>CH_4: 2.2 μmol g^{-1} h^{-1}		
$Pt_{1.5\%}$–0.50–graphene/ reduced titania	CO_2/water vapor	C_2H_6: 6.7 μmol g^{-1} h^{-1}<br>CH_4: 17.4 μmol g^{-1} h^{-1}	27.9 [b]	22
Au NPs	CO_2-saturated IPA aqueous solution	C_2H_6: N/A<br>CH_4: N/A	~ 40 [b]	23
$Cu^{\delta+}$/CeO_2-TiO_2	CO_2-saturated water	C_2H_4: 4.51 μmol g^{-1} h^{-1}<br>CO: 3.47 μmol g^{-1} h^{-1}<br>CH_4: 1.52 μmol g^{-1} h^{-1}	73.9 [a]	24
Partially reduced Co_3O_4	CO_2/water vapor	CH_3COOH: 2.95 μmol g^{-1} h^{-1}<br>CO: 0.95 μmol g^{-1} h^{-1}	92.5 [a]	25
V_O-rich Zn_2GeO_4	Simulated air in water	CH_3COOH: 12.7 μmol g^{-1} h^{-1}<br>CO: ~13.5 μmol g^{-1} h^{-1}<br>HCOOH: ~11.5 μmol g^{-1} h^{-1}	66.9 [a]	26
Magnetic-field-regulated TiO_2	CO_2 -saturated $NaHCO_3$ aqueous solution	CH_3CH_2OH: 6.16 μmol g^{-1} h^{-1}<br>CH_4: 1.60 μmol g^{-1} h^{-1}<br>CO: 0.82 μmol g^{-1} h^{-1}<br>CH_3OH: 0.34 μmol g^{-1} h^{-1}<br>HCOOH: 0.12 μmol g^{-1} h^{-1}	66.71 [b]	27
V_S-$AgInP_2S_6$ SAL	CO_2/water vapor	C_2H_4: 44.3 μmol g^{-1} (1st h)<br>CO: 10.9 μmol g^{-1} (1st h)<br>CH_4: 5.6 μmol g^{-1} (1st h)	89 [a]	28
V_O-rich WO_3·0.33H_2O	CO_2-saturated water	CH_3COOH: 9.4 μmol g^{-1} h^{-1}<br>HCOOH: 1.5 μmol g^{-1} h^{-1}<br>CO: 0.112 μmol g^{-1} h^{-1}	85 [b]	29
$Cu_{1.00\%}$–$Pt_{0.35\%}$–blue Titania	CO_2/water vapor	C_2H_6: 0.15 mmol g^{-1} (6 h)<br>CH_4: 3 mmol g^{-1} (6 h)	4.8 [b]	30
d-UiO-66/MoS_2-5 wt %	CO_2-saturated water	CH_3COOH: 39.0 μmol g^{-1} h^{-1}<br>CH_3CH_2OH: 2.5μmol g^{-1} h^{-1}	94 [b]<br>6 [b]	31
Cu SAs/UiO-66-NH_2	CO_2-saturated water	CH_3CH_2OH: 4.22 μmol g^{-1} h^{-1}<br>CH_3OH: 5.33 μmol g^{-1} h^{-1}	44.2 [b]	32
C-SnS_2	CO_2/water vapor	CH_3CHO: 0.09 μmol cm^{-2} h^{-1}	< 15 [b]	33

		CH_4: 0.75 μmol cm^{-2} h^{-1}		
C/Cu_2O	CO_2-saturated $KHCO_3$ aqueous solution	C_2H_4: ~0.22 μmol (12 h)<br>CH_4: ~0.16 μmol (12 h)	~ 58 [b]	[34]
Cu/GO	CO_2/water vapor	CH_3CHO: 3.88 μmol g^{-1} h^{-1}<br>CH_3OH: 2.94 μmol g^{-1} h^{-1}	56.9 [b]	[35]
Graphene-TiO_2	CO_2/water vapor	C_2H_6: 16.8 μmol g^{-1} h^{-1}<br>CH_4: 8 μmol g^{-1} h^{-1}<br>CO: 2.3 μmol g^{-1} h^{-1}	67.7 [b]	[36]

[a] Electron-based selectivity. [b] Product-based selectivity.

Supplementary Table 4. Comparison for the ratio of products at 5 h/ 3h using $^{13}CO_2$ and $^{12}CO_2$ as feed-gas.

	$^{13}CO_2$ as feed-gas (Fig. S21)			$^{12}CO_2$ as feed-gas (Fig. 3a)	
Product	MS peak area at 3 h	MS peak area at 5 h	Ratio of MS peak area (5 h/ 3h)	Ratio of gas evolution amount (5 h/ 3h)	Difference (%)
C_3H_8	174.092	296.927	1.706	1.877	9.1
C_2H_4	72.252	121.007	1.675	1.832	8.6
CO	175.367	296.649	1.692	1.842	8.2

The slight difference of the products generation for $^{12}CO_2$ and $^{13}CO_2$ feed-gas is probably due to the different sensitivity of signal response of GC and GC-MS caused by the different detectors and operating conditions.

Supplementary Note 1. In-situ DRIFTS spectra analysis of Cu-Ti-V_O/$Ti_{0.91}O_2$-SL.

In-situ diffuse reflectance Fourier transform infrared spectroscopy (DRIFTS) measurement was performed to identify the reaction intermediates of the CO_2 photoreduction process. The strong band at 1650 cm^{-1} is ascribed to the surface adsorbed water. The band around 1691 and 1254 cm^{-1} is attributed to the absorbed $*CO_2^-$ species[37-41]. The bands at 1378 and 1482 cm^{-1} are associated with the symmetric vibration and C-O stretching of *COOH group, a key intermediate for the formation of CO, respectively[42,43]. The bands at 2077 cm^{-1} correspond to the chemical bound *CO[44]. These results indicate that the surface *CO is generated as an important intermediate. Besides, bands at 1461 cm^{-1} and 1584 cm^{-1} are ascribed to *CHO[21] and *COCHO[42] intermediate, respectively, revealing that the first C-C coupling at Cu-Ti-V_O units probably takes place between *CHO and the migrating CO[45].

The in-situ DRIFTS measurement was also carried out with isotope labelling, using $^{13}CO_2$ to replace $^{12}CO_2$. The spectra are shown in Supplementary Fig. 25. All the bands of carbon-containing intermediates are observed when using $^{13}CO_2$, and their band positions shift to lower wavenumber compared with that of $^{12}CO_2$, indicating that these bands are indeed from the intermediates of CO_2 reduction reaction rather than the surface impurities.

Supplementary Note 2. The calculated reaction pathway on $Ti_{0.91}O_2$ matrix.

On $Ti_{0.91}O_2$ matrix domain, CO_2 is reduced to *CO through *COOH intermediate. After *CO is formed on $Ti_{0.91}O_2$ matrix, the Gibbs free energy changes of different reaction routes are considered, including the desorption of *CO, the hydrogenation of *CO and the C-C coupling. The desorption energy of *CO on $Ti_{0.91}O_2$ matrix is estimated to be -0.29 eV. The free energy change for the hydrogenation of *CO is 0.27 eV. For C_1-C_1 coupling, the free energy changes of the *CO-CO or *CHO-CO coupling is 1.31 or 0.37 eV. As for the C_1-C_2 coupling, the potential *C_3 intermediate (*CH_2OCOCO) cannot exist as a stable molecule on $Ti_{0.91}O_2$ matrix according to DFT computation results, indicating that C_1-C_2 coupling is forbidden on $Ti_{0.91}O_2$ matrix. Thus, the desorption of *CO is more preferred on $Ti_{0.91}O_2$ matrix, while hydrogenation of *CO or C-C coupling is hindered. The calculated results are consistent with the photocatalytic performance of pristine $Ti_{0.91}O_2$-SL where CO is generated as the dominant final product.

Supplementary Note 3. The calculated C_3H_8 formation pathway on Cu-Ti-V_O unit.

According to the previous literatures, the most-reported potential reaction mechanisms for C_{2+} production include *CO-CO[46-51] (*COCO-CO[47,52-54]), *COH-CO[55-57], and *CHO-CO[42,58-60] pathways. We chose the *CHO-CO pathway in this work instead of CO* dimerization to *OCCO because our DRIFTS characterization has detected the formation of the *CHOCO intermediates on Cu-Ti-V_O/$Ti_{0.91}O_2$-SL. For C_1 key intermediate, the *CHO pathway is selected since proton–electron transfer to CO* via the CHO pathway is lower 0.55 eV than that of the COH pathway[56]. Thus, the C_{2+} intermediates *COCOH formed by *CO and *COH is less-favorable than *COCHO. We also studied other possible reaction pathways on Cu-Ti-V_O units, namely the hydrogenation of *CHO and *CH_2OCO, respectively. The energy change of the hydrogenation of *CHO is -0.23eV, while the energy change of the coupling between *CHO and CO is -0.5eV, indicating that the hydrogenation of *CHO is less preferred than *CHO-CO coupling. The energy change of the hydrogenation of *CH_2OCO to *CH_2OCHO is -0.73 eV, more negative than the -0.13eV energy change of *CH_2OCO-CO. However, the *CH_2OCHO-CO has a huge energy barrier of 0.62 eV, much higher than the *CH_2OCO-CO pathway. It should be noted that considering the complexity of the formation mechanism of the C_{2+} products, we give here only the most probable results for CO_2 reduction to propane on Cu-Ti-V_O units based on experimental results and theoretical calculations. With ever-increasing computing power and rapid development of in-situ characterization techniques, it is believed that a more comprehensive and universal analysis of the C_{2+} mechanism can be given in the future.

Supplementary Note 4. The calculated reaction pathway on Cu-O site.

*CO is first generated at Cu-O site through *COOH intermediate, which are hydrogenated to *CHO intermediates with unsaturated coordination. The formed *CHO tends to further hydrogenate to *CHOH with a negative free energy change of -1.47 eV and is eventually reduced to CH_4 through multistep hydrogenation. However, C-C coupling is thermodynamically unfavourable at Cu-O sites, as the first coupling between *CHO and *CO involves an uphill energy change of 0.17 eV, and the second coupling between *CH_2OCO and *CO has a higher energy barrier of 1.85 eV. The calculated results match well with the experimental observation of Cu-O/$Ti_{0.91}O_2$-SL.

Supplementary References

1 Song, Y. et al. Photocatalytic hydrogen evolution over monolayer $H_{1.07}Ti_{1.73}O_4 \cdot H_2O$ nanosheets: Roles of metal defects and greatly enhanced performances. *Appl. Catal. B: Environ.* **221**, 473-481 (2018).

2 Ge, H. et al. Photocatalytic conversion of CO_2 into light olefins over TiO_2 nanotube confined Cu clusters with high ratio of Cu^+. *Appl. Catal. B: Environ.* **263,** 118133 (2020).

3 Wang, J. et al. A Single Cu-center containing enzyme-mimic enabling full photosynthesis under CO_2 reduction. *ACS Nano* **14**, 8584-8593 (2020).

4 Liu, A. et al. Controlling dynamic structural transformation of atomically dispersed CuO_x Species and influence on their catalytic performances. *ACS Catal.* **9**, 9840-9851 (2019).

5 Huang, F. et al. Anchoring Cu_1 species over nanodiamond-graphene for semi-hydrogenation of acetylene. *Nat. Commun.* **10**, 4431 (2019).

6 Zhang, F.-F. et al. Iridium oxide modified with silver single stom for boosting oxygen evolution reaction in acidic media. *ACS Energy Lett.* **6**, 1588-1595 (2021).

7 Xiao, M. et al. Molten-salt-mediated synthesis of an atomic nickel co-catalyst on TiO_2 for improved photocatalytic H_2 evolution. *Angew. Chem. Int. Ed.* **59**, 7230-7234 (2020).

8 Chen, Y. et al. Engineering the atomic interface with single platinum atoms for enhanced photocatalytic hydrogen production. *Angew. Chem. Int. Ed.* **59**, 1295-1301 (2020).

9 Zhang, G. *et al.* Soluble complexes of cobalt oxide fragments bring the unique CO_2 photoreduction activity of a bulk material into the flexible domain of molecular science. *J. Am. Chem. Soc.* **143**, 20769-20778 (2021).

10 van Deelen, T. W., Hernández Mejía, C. & de Jong, K. P. Control of metal-support interactions in heterogeneous catalysts to enhance activity and selectivity. *Nat. Catal.* **2**, 955-970 (2019).

11 Guo, Y. et al. Low-temperature CO_2 methanation over CeO_2-supported Ru single atoms, nanoclusters, and nanoparticles competitively tuned by strong metal–support interactions and H-spillover effect. *ACS Catal.* **8**, 6203-6215 (2018).

12 Atrei, A., Ferrari, A. M., Szieberth, D., Cortigiani, B. & Rovida, G. Lepidocrocite-like structure of the TiO_2 monolayer grown on Ag(100). *Phys. Chem. Chem. Phys.* **12**, 11587-11595 (2010).

13 Zhang, G. et al. Soluble complexes of cobalt oxide fragments bring the unique CO_2 photoreduction activity of a bulk material into the flexible domain of molecular science. *J. Am. Chem. Soc.* **143**, 20769-20778 (2021).

14 Ou, M. et al. Amino-assisted anchoring of $CsPbBr_3$ perovskite quantum dots on porous g-C_3N_4 for enhanced photocatalytic CO_2 reduction. *Angew. Chem. Int. Ed.* **57**, 13570-13574 (2018).

15 Li, J. et al. Self-adaptive dual-metal-site pairs in metal-organic frameworks for selective CO_2 photoreduction to CH_4. *Nat. Catal.* **4**, 719-729 (2021).

16 Qian, G. et al. Efficient photoreduction of diluted CO_2 to tunable syngas by Ni-Co dual sites through d-band center manipulation. *Angew Chem. Int. Ed.* **61**, e202210576 (2022).

17 Han, C. et al. Cooperative syngas production and C-N bond formation in one photoredox cycle. *Angew Chem. Int. Ed.* **60**, 7962-7970 (2021).

18 Yang, J. et al. In-situ polymerization induced atomically dispersed manganese sites as cocatalyst for CO_2 photoreduction into synthesis gas. *Nano Energy* **76**, 105059 (2020).

19 Yu, S. & Jain, P. K. Plasmonic photosynthesis of C_1-C_3 hydrocarbons from carbon dioxide assisted by an ionic liquid. *Nat. Commun.* **10**, 2022 (2019).

20 Li, N. et al. Toward high-value hydrocarbon generation by photocatalytic reduction of CO_2 in water vapor. *ACS Catal.* **9**, 5590-5602 (2019).

21 Wang, W. et al. Photocatalytic C-C coupling from carbon dioxide reduction on copper oxide with mixed-valence

copper(I)/copper(II). *J. Am. Chem. Soc.* **143**, 2984-2993 (2021).

22 Sorcar, S. et al. High-rate solar-light photoconversion of CO_2 to fuel: Controllable transformation from C_1 to C_2 products. *Energy Environ. Sci.* **11**, 3183 (2018).

23 Yu, S., Wilson, A. J., Heo, J. & Jain, P. K. Plasmonic control of multi-electron transfer and C-C coupling in visible-light-driven CO_2 reduction on Au nanoparticles. *Nano Lett.* **18**, 2189-2194 (2018).

24 Wang, T. et al. Engineering catalytic interfaces in $Cu^{\delta+}/CeO_2$-TiO_2 photocatalysts for synergistically boosting CO_2 reduction to ethylene. *ACS Nano* **16**, 2306-2318 (2022).

25 Zhu, S. et al. Selective CO_2 photoreduction into C_2 product enabled by charge-polarized metal pair sites. *Nano Lett.* **21**, 2324-2331 (2021).

26 Zhu, J. et al. Asymmetric triple-atom sites confined in ternary oxide enabling selective CO_2 photothermal reduction to acetate. *J. Am. Chem. Soc.* **143**, 18233-18241 (2021).

27 Jiang, M.-P. et al. Magnetic-field-regulated TiO_2 {100} facets: A strategy for C-C coupling in CO_2 photocatalytic conversion. *Chem* **6**, 2335-2346 (2020).

28 Gao, W. et al. Vacancy-defect modulated pathway of photoreduction of CO_2 on single atomically thin $AgInP_2S_6$ sheets into olefiant gas. *Nat. Commun.* **12**, 4747 (2021).

29 Sun, S., Watanabe, M., Wu, J., An, Q. & Ishihara, T. Ultrathin WO_3.0.33H_2O nanotubes for CO_2 photoreduction to acetate with high selectivity. *J. Am. Chem. Soc.* **140**, 6474-6482 (2018).

30 Sorcar, S. et al. CO_2, water, and sunlight to hydrocarbon fuels: a sustained sunlight to fuel (Joule-to-Joule) photoconversion efficiency of 1%. *Energy Environ. Sci.* **12**, 2685-2696 (2019).

31 Yu, F., Jing, X., Wang, Y., Sun, M. & Duan, C. Hierarchically porous metal-organic framework/MoS_2 interface for selective photocatalytic conversion of CO_2 with H_2O into CH_3COOH. *Angew. Chem. Int. Ed.* **60**, 24849-24853 (2021).

32 Wang, G. et al. Photoinduction of Cu single atoms decorated on UiO-66-NH_2 for enhanced photocatalytic reduction of CO_2 to liquid fuels. *J. Am. Chem. Soc.* **142**, 19339-19345 (2020).

33 Billo, T. et al. A mechanistic study of molecular CO_2 interaction and adsorption on carbon implanted SnS_2 thin film for photocatalytic CO_2 reduction activity. *Nano Energy* **72**, 104717 (2020).

34 Yu, L. et al. Enhanced activity and stability of carbon-decorated cuprous oxide mesoporous nanorods for CO_2 reduction in artificial photosynthesis. *ACS Catal.* **6**, 6444-6454 (2016).

35 Shown, I. et al. Highly efficient visible light photocatalytic reduction of CO_2 to hydrocarbon fuels by Cu-nanoparticle decorated graphene oxide. *Nano Lett.* **14**, 6097-6103 (2014).

36 Tu, W. et al. An in situ simultaneous reduction-hydrolysis technique for fabrication of TiO_2-graphene 2D sandwich-like hybrid nanosheets: Graphene-promoted selectivity of photocatalytic-driven hydrogenation and coupling of CO_2 into methane and ethane. *Adv. Funct. Mater.* **23**, 1743-1749 (2013).

37 Shi, X. et al. Photoswitchable chlorine vacancies in ultrathin $Bi_4O_5Cl_2$ for selective CO_2 photoreduction. *ACS Catal.* **12**, 3965-3973 (2022).

38 Sheng, J. *et al.* Identification of halogen-associated active sites on bismuth-based perovskite quantum dots for efficient and selective CO_2-to-CO photoreduction. *ACS Nano* **14**, 13103-13114 (2020).

39 Cheng, L., Zhang, D., Liao, Y., Fan, J. & Xiang, Q. Structural engineering of 3D hierarchical $Cd_{0.8}Zn_{0.2}S$ for selective photocatalytic CO_2 reduction. *Chinese J. Catal.* **42**, 131-140 (2021).

40 Liu, L. *et al.* Engineering coexposed {001} and {101} facets in oxygen-deficient TiO_2 nanocrystals for enhanced CO_2 photoreduction under visible light. *ACS Catal.* **6**, 1097-1108 (2016).

41 Wang, Y. *et al.* CO_2 photoreduction with H_2O vapor on highly dispersed CeO_2/TiO_2 catalysts: Surface species and their reactivity. *J. Catal.* **337**, 293-302 (2016).

42 Qiu, X. F., Zhu, H. L., Huang, J. R., Liao, P. Q. & Chen, X. M. Highly selective CO_2 electroreduction to C_2H_4

using a metal-organic framework with dual active sites. *J. Am. Chem. Soc.* **143**, 7242-7246 (2021).

43 Chou, T. C. et al. Controlling the oxidation state of the Cu electrode and reaction intermediates for electrochemical CO_2 reduction to ethylene. *J. Am. Chem. Soc.* **142**, 2857-2867 (2020).

44 Wang, M. et al. Oxygen vacancy generation and stabilization in CeO_{2-x} by Cu introduction with improved CO_2 photocatalytic reduction activity. *ACS Catal.* **9**, 4573-4581 (2019).

45 Ma, W. et al. Electrocatalytic reduction of CO_2 to ethylene and ethanol through hydrogen-assisted C–C coupling over fluorine-modified copper. *Nat. Catal.* **3**, 478-487 (2020).

46 Ma, Z. et al. CO_2 electroreduction to multicarbon products in strongly acidic electrolyte via synergistically modulating the local microenvironment. *Nat. Commun.* **13**, 7596 (2022).

47 Wang, X. et al. Efficient upgrading of CO to C_3 fuel using asymmetric C-C coupling active sites. *Nat. Commun.* **10**, 5186 (2019).

48 Liang, Z. Q. et al. Copper-on-nitride enhances the stable electrosynthesis of multi-carbon products from CO_2. *Nat. Commun.* **9**, 3828 (2018).

49 Xie, Y. et al. High carbon utilization in CO_2 reduction to multi-carbon products in acidic media. *Nat. Catal.* **5**, 564-570 (2022).

50 Cheng, T., Xiao, H. & Goddard, W. A., 3rd. Full atomistic reaction mechanism with kinetics for CO reduction on Cu(100) from ab initio molecular dynamics free-energy calculations at 298 K. *Proc. Natl. Acad. Sci.* **114**, 1795-1800 (2017).

51 Calle-Vallejo, F. & Koper, M. T. Theoretical considerations on the electroreduction of CO to C_2 species on Cu(100) electrodes. *Angew. Chem. Int. Ed.* **52**, 7282-7285 (2013).

52 Zhuang, T.-T. et al. Copper nanocavities confine intermediates for efficient electrosynthesis of C_3 alcohol fuels from carbon monoxide. *Nat. Catal.* **1**, 946-951 (2018).

53 Peng, C. et al. Double sulfur vacancies by lithium tuning enhance CO_2 electroreduction to n-propanol. *Nat. Commun.* **12**, 1580 (2021).

54 Hu, F. et al. Ultrastable Cu catalyst for CO_2 electroreduction to multicarbon liquid fuels by tuning C-C coupling with CuTi subsurface. *Angew. Chem. Int. Ed.* **60**, 26122-26127 (2021).

55 Das, K. et al. Intrinsic charge polarization in $Bi_{19}S_{27}Cl_3$ nanorods promotes selective C-C coupling reaction during photoreduction of CO_2 to ethanol. *Adv. Mater.* e2205994 (2022).

56 Calle-Vallejo, F. & Koper, M. T. M. Accounting for bifurcating pathways in the screening for CO_2 reduction catalysts. *ACS Catal.* **7**, 7346-7351 (2017).

57 Perez-Gallent, E., Figueiredo, M. C., Calle-Vallejo, F. & Koper, M. T. Spectroscopic observation of a hydrogenated CO dimer intermediate during CO reduction on Cu(100) electrodes. *Angew. Chem. Int. Ed.* **56**, 3621-3624 (2017).

58 Ou, H. et al. Atomically dispersed au-assisted C-C coupling on red phosphorus for CO_2 photoreduction to C_2H_6. *J. Am. Chem. Soc.* **144**, 22075-22082 (2022).

59 Liu, Q. et al. Regulating the *OCCHO intermediate pathway towards highly selective photocatalytic CO_2 reduction to CH_3CHO over locally crystallized carbon nitride. *Energy Environ. Sci.* **15**, 225-233 (2022).

60 Garza, A. J., Bell, A. T. & Head-Gordon, M. Mechanism of CO_2 reduction at copper surfaces: Pathways to C_2 products. *ACS Catal.* **8**, 1490-1499 (2018).
